# Effect of an Outdoor-Focused Licensed Child Care Program on Child, Caregiver, and Educator Outcomes, Inclusion, and Accessibility: Protocol for the Sending Preschoolers Outside (SPROUT) Prospective Cohort Study

**DOI:** 10.2196/89405

**Published:** 2026-07-21

**Authors:** Maeghan James, Louise de Lannoy, Kelly P Arbour-Nicitopoulos, Alessia Capone, Lisa Belton, Kim Hiscott, Lisa Lalonde, Tammy Potter, Paula ter Huurne, Mark S Tremblay

**Affiliations:** 1 School of Epidemiology and Public Health, Faculty of Medicine, University of Ottawa, 600 Peter Morand, Ottawa, Ontario, K1G 5Z3, Canada, 1 (613) 737-7600; 2 Health Active Living and Obesity Research Group, Children's Hospital of Eastern Ontario Research Institute, Ottawa, Ontario, Canada; 3 Outdoor Play Canada, Ottawa, Ontario, Canada; 4 Faculty of Kinesiology and Physical Education, University of Toronto, Toronto, Ontario, Canada; 5 Andrew Fleck Children’s Services, Ottawa, Ontario, Canada; 6 Public Safety & Community Studies, School of Wellness, Algonquin College, Ottawa, Ontario, Canada

**Keywords:** early childhood, child health, child development, public health intervention, outdoor play, childhood disability

## Abstract

**Background:**

Outdoor play supports children’s physical, social, and cognitive development; yet, opportunities are declining as indoor, screen-based childhoods become the norm. Early Learning and Child Care (ELCC) centers are key settings for promoting outdoor play, but limited evidence exists on the health and developmental outcomes of licensed nature-based ELCC programs, particularly for children with and those without disabilities.

**Objective:**

The Sending Preschoolers Outside (SPROUT) and SPROUT-able studies aim to examine the impact of one of Canada’s first purposely designed and built licensed outdoor-focused ELCC programs. The primary objective is to examine the health and developmental outcomes of children aged 10‐59 months, with and those without disabilities, attending an outdoor-focused, full-day licensed ELCC program compared to those in conventional (eg, not outdoor-focused) ELCC settings. Secondary objectives are to (1) examine educator job satisfaction, health, well-being, and attitudes toward teaching outdoors for children with and those without disabilities; (2) examine caregiver beliefs and attitudes toward children’s participation in outdoor play and learning; and (3) identify barriers and facilitators to outdoor play and learning for children with and those without disabilities (ie, the SPROUT-able substudy) in an outdoor-focused ELCC program.

**Methods:**

This prospective longitudinal open cohort study follows children, caregivers, and educators over 3 years. Participants are recruited from 1 outdoor-focused (experimental) and 3 conventional (usual care) ELCC centers in Ontario, Canada; all sites are licensed. Assessments occur every 6 months and include direct measures of children (eg, anthropometry, motor skills, and accelerometry) and caregiver and educator questionnaires. Mixed-effects modeling will examine changes over time and between groups (experimental vs usual care).

**Results:**

As of August 2025, 86 caregiver-child dyads are enrolled in the study (67 experimental and 43 control). Of these, 70 children who enrolled at study initiation have completed baseline and T1 and T2 assessments (43 experimental and 27 usual care), while 18 children enrolled at the 6-month time point (T1; 13 experimental and 5 usual care) have completed T1 and T2 (with a missing baseline assessment). A total of 19 children from the experimental group were identified as having a disability meeting criteria for the SPROUT-able substudy: 4 with a formal diagnosis, 3 with caregiver-reported functional difficulties, and 12 receiving additional support from an inclusion support worker.

**Conclusions:**

By combining a holistic and comparative approach with a focus on children with and those without disabilities, the SPROUT and SPROUT-able studies will generate comprehensive evidence to inform ELCC practice and policy in Canada and internationally. This model within Canada provides timely evidence on outdoor-focused ELCC delivery, integrating disability inclusive outdoor play into ELCC settings, and has the potential to guide meaningful improvements in child development, educator well-being, and community health.

## Introduction

Outdoor play is essential for healthy child development, with evidence of positive changes in physical, mental, and social-emotional health [1-4]. However, societal changes such as screen-based entertainment and rising concerns over children’s safety have led to more time spent indoors and less outdoor play [3]. With more than half of children in Canada attending licensed Early Learning and Child Care (ELCC) programs [5], outdoor-focused ELCC programs (ie, licensed ELCC programs that prioritize outdoor and nature play, beyond the minimum licensing requirement) provide an opportunity to reintroduce outdoor play in early childhood. Although children remain the central focus of such programs, evidence suggests that engaging in outdoor play also benefits adults’ physical and mental health [4]. As a result, outdoor-focused ELCC programs may offer a strategy to support early childhood educators’ (herein referred to as “educators”) well-being. Emerging findings from the United Kingdom indicate that outdoor-focused programs are associated with improved educator well-being and professional engagement and development [6], suggesting potential contributions to educator retention, job satisfaction, and the overall quality of care. Taken together, the potential benefits for both children and educators reinforce the value of outdoor play in ELCC programs and the need to increase the availability of programs that support access to outdoor play in nature.

In Ontario, Canada, licensed ELCC programs are required to provide a minimum of 2 hours of outdoor play per day for children in 6 or more hours of care, weather permitting, unless otherwise exempted by a caregiver (biological or nonbiological, as well as any adult who serves as the child’s primary caregiver) or physician [7]. Despite this requirement, a recent systematic review and meta-analysis found that across 26 studies reporting on outdoor time in conventional ELCC centers (ie, licensed center-based ELCC programs primarily delivered indoors), children spent on average 45 minutes outdoors, with some programs reporting as little as 10 minutes per day [8]. The prioritization of indoor over outdoor time in ELCC settings is problematic, as research shows children are more active and less sedentary when outdoors [9], contributing to positive health and developmental benefits. It is possible that indoor time is prioritized in ELCC settings due to the perception that quality learning can only occur inside, despite a growing body of evidence to the contrary [10], and that outdoor time is for leisure pursuits only [11]. Other factors, such as temperature, climate, air quality, accessibility, perceived danger, licensing restrictions, and lack of preservice training in outdoor play, may further discourage educators from bringing children outdoors for prolonged periods of time. Given the potential benefits of outdoor time and play for children’s and educators’ overall health and well-being, efforts are needed to challenge preconceived ideas about the outdoors and promote outdoor play and learning in ELCC programs.

Outdoor play during ELCC hours is even less common for children with disabilities. For example, in a study conducted in the United States, children with disabilities spent 80% of their time at ELCC indoors, with most of their indoor activity being sedentary [12]. For children with disabilities, outdoor built and social environments are often designed in ways that limit their participation in active play [13-16]. Barriers in the built and social environments in the outdoors result in children with disabilities being excluded from outdoor play and learning experiences. Further, caregivers of children with disabilities may feel hesitant to have their child engage in outdoor play due to the inherent or perceived risk [17,18]. Spending time outdoors is beneficial to all children, including children with disabilities [1,2,19], and therefore, it is critical that efforts seeking to increase outdoor-focused ELCC programs are inclusive and accessible to children with and those without disabilities attending ELCC.

Shifting the overall mindset about outdoor play and learning for children with and those without disabilities requires a stronger evidence base to support outdoor-focused ELCC programs. Research on outdoor-focused ELCC is sparse; however, preliminary findings from an American pilot program suggest that outdoor-focused ELCC programs equally prepare children for school while also facilitating more diverse learning and skill acquisition, compared to conventional ELCC programs [20,21]. More robust research, and within a Canadian context, is required to confirm these preliminary observations and to determine the efficacy of outdoor-focused ELCC programs for promoting healthy child development and learning, educator health and well-being, and the relative benefits and risks associated with outdoor play for children with and those without disabilities.

The Sending Preschoolers Outside (SPROUT) study is a 3-year, multicomponent study that seeks to compare an outdoor-focused ELCC program to conventional non–outdoor-focused ELCC programs in Ontario, Canada. It is designed to understand whether and how outdoor-focused ELCC programs contribute to child health and development and to explore the potential benefits to educators and families. The primary objective of this study is to examine the relationship between attending an outdoor-focused, full-day licensed ELCC program and the physical, cognitive, and social-emotional development of children with and those without disabilities aged 10‐59 months, compared to children attending conventional ELCC programs. The secondary objectives of this study are to (1) examine educator job satisfaction, health and well-being, and beliefs and attitudes toward teaching in outdoor environments for children with and those without disabilities between the 2 types of ELCC programs; (2) examine caregiver beliefs and attitudes toward children’s participation in outdoor play and learning between the 2 types of programs; and (3) understand the barriers and facilitators to outdoor play and learning at an outdoor-focused ELCC center for children with and those without disabilities through the substudy “SPROUT-able.”

## Methods

### Study Design

This study uses a 3-year, prospective longitudinal (open) cohort design to compare (1) child development (physical, social-emotional, and cognitive) and children’s movement behaviors; (2) educator health and well-being; (3) educator job satisfaction and attitudes and beliefs toward outdoor play and learning; and (4) caregivers’ attitudes and beliefs toward outdoor play and learning between those at an outdoor-focused versus conventional full-day licensed ELCC program. We use a continuous recruitment approach (open cohort), whereby families and educators are invited to participate in the study leading up to either of the 2 annual data collection time points [22].

### Ethical Considerations

**Figure 1.**
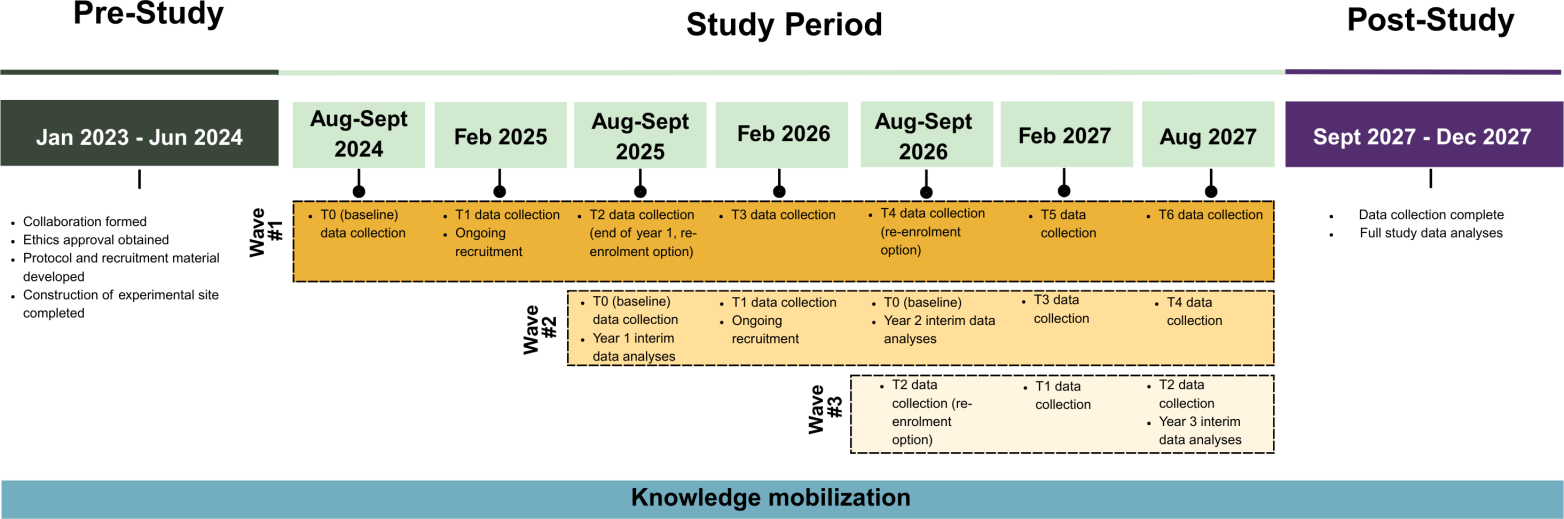
SPROUT (Sending Preschoolers Outside) study timeline.

This study has received institutional research ethics board approval (#23/108X). Caregiver-child dyads and educators consent to 1 year of the study and are encouraged to reenroll for up to 2 years as long as the child remains at the center (refer to Figure 1 for study timeline).

### Participants and Settings

Caregiver-child dyads are eligible to participate if children are between the ages of 10‐59 months at baseline, attend a participating ELCC center for 6 or more hours per day, 3 or more days per week, and if the caregiver can read, write, and understand English and/or French. Children with and those without disabilities will be eligible to participate in this study and efforts will be made to ensure study procedures and instruments fit the needs and abilities of children with disabilities. Educators are eligible if they are currently employed at a participating ELCC center, and they can read, write, and understand English and/or French. Participants are recruited from 2 types of ELCC programs: outdoor-focused (experimental) and conventional programs (usual care). The experimental and usual care programs are both governed by the same childcare organization and are licensed under the same provincial regulations. Given this, the centers are comparable in terms of cost per day, staffing ratios, and mealtime and nap protocols. The main difference between the centers lies in how the centers deliver the early years curriculum and where programming takes place. Details of the experimental and conventional centers’ programs, including setting, curriculum, and time outside, are detailed below.

The outdoor-focused ELCC center serves as the experimental group. This center prioritizes extended outdoor play and learning, with children spending the majority of the day outside year-round, regardless of the weather. The center is situated on 500 acres of forested land that children explore daily for learning and play. This expansive natural environment features a thick canopy of tall, established trees that provide significant shade, as well as a rich and diverse landscape with largely natural, grassy ground cover. Tree species are well mixed and include native varieties such as sugar maples, oaks, and several coniferous species. The terrain is varied, with exposed roots, rocky sections, and small but steep elevation changes, alongside wetlands and visible rock deposits that contribute to the site’s natural character and afford diverse sensory and physical experiences.

**Figure 2.**
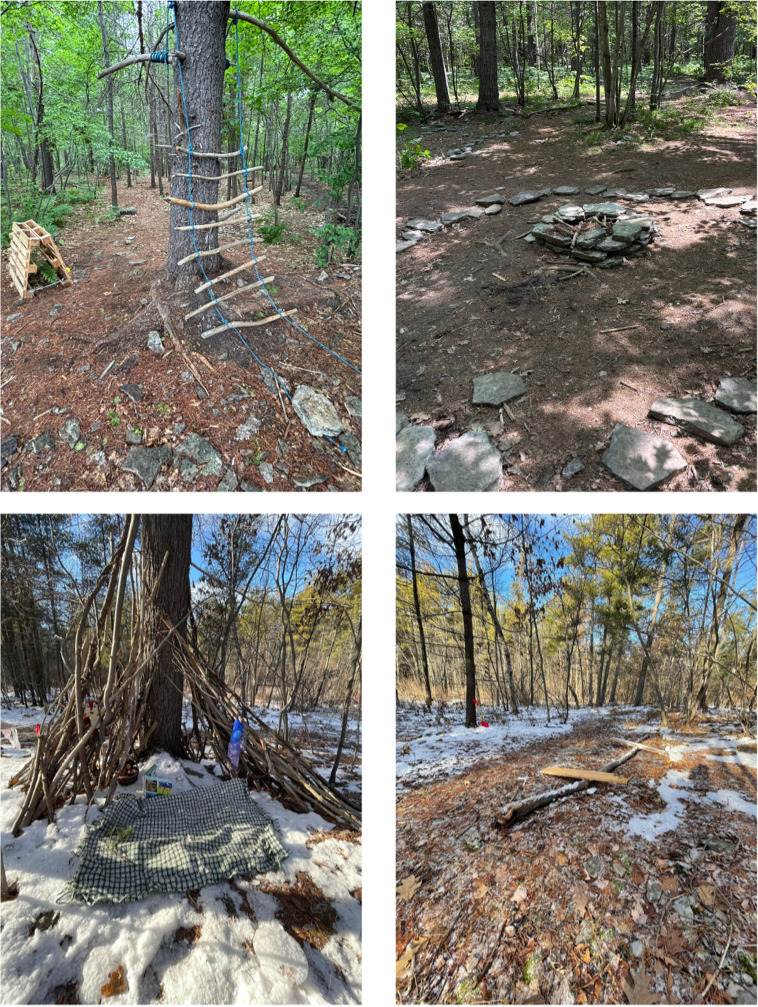
Images of the outdoor space at the outdoor-focused Early Learning and Child Care program (ie, experimental site).

Each class has a designated “outdoor learning space” within the forested area. The infant group (<18 months) outdoor space is located closer to the building than those of the toddler (18‐30 months) and preschool (30‐71 months) groups; however, all classes have the flexibility to move beyond these areas depending on weather conditions and children’s emerging interests (Figure 2). Educators establish strong relationships with children on the land, setting clear boundaries that support safe supervision while still allowing for autonomy and exploration. The outdoor learning environments are intentionally designed to support inquiry-based and play-based learning. Natural and thoughtfully introduced provocations are embedded throughout the forest to spark curiosity and investigation. Features of the outdoor space include a fire circle, a sheltered eating area, picnic tables, climbing structures (eg, pallets and rope ladders), a secondary stick-built structure, and a rock wall that helps define boundaries. Daily experiences often include mud kitchen play, water and puddle exploration, gardening, and searching for insects and other small creatures (eg, salamanders, worms, and slugs). Natural materials such as sticks, rocks, and plants play a central role in children’s play and learning, supporting creativity, problem-solving, and physical engagement. The curriculum, while guided by the provincial early learning framework [22], emphasizes exploration, creativity, resilience, and environmental stewardship through immersive experiences in nature. Children are encouraged to engage with natural materials, ask questions, and observe ecological processes, such as plant growth and seasonal changes. Learning is child-led, play-based, and developmentally appropriate, with a strong emphasis on sensory experiences, movement, and connection to the natural world. Educators at the outdoor-focused ELCC typically hold specialized training, such as the Child and Nature Alliance of Canada’s Forest Practitioner certification or the Algonquin College Play, Learn and Teach Outdoors certificate. The educator team supports extended outdoor engagement through practices such as outdoor meals and snacks when feasible, as well as outdoor diapering routines for younger children. Indoor environments are used primarily for meals, rest, transitions, and as a safe refuge during extreme weather conditions (eg, excessive heat, severe cold, or thunderstorms).

**Figure 3.**
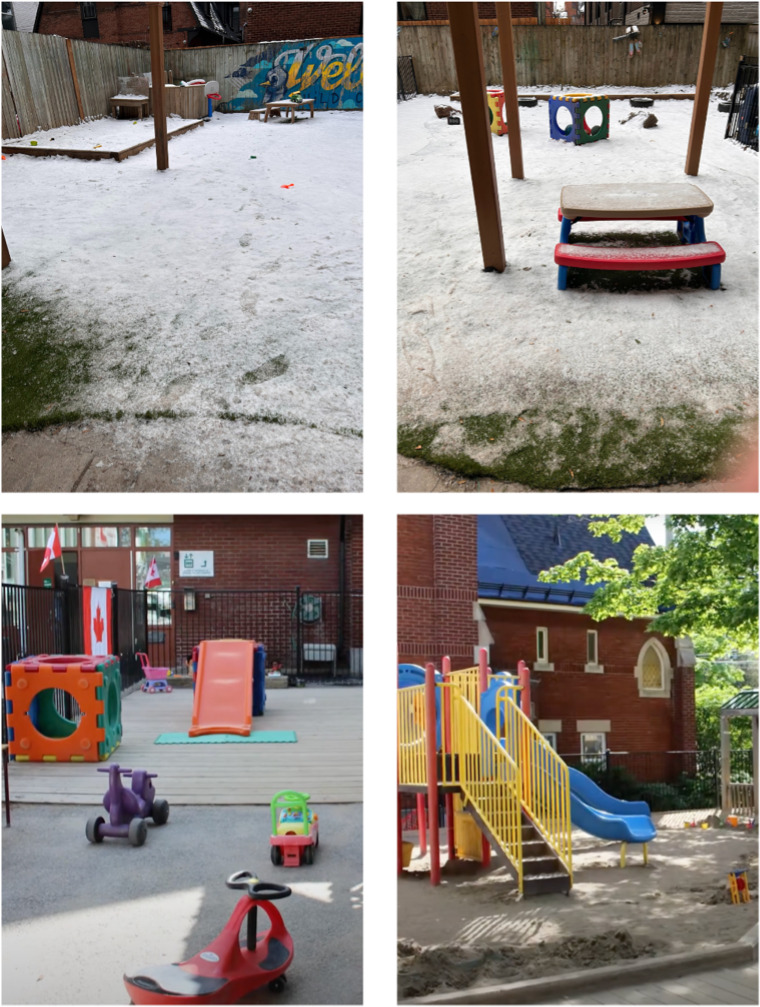
Images of the outdoor space at the conventional Early Learning and Child Care programs (ie, usual care sites).

Three conventional ELCC centers will serve as the usual care control group. These centers provide conventional programming with scheduled outdoor time typically meeting the regulated amount of outdoor time, a minimum of 2 hours a day (eg, 60 minutes, twice daily) in defined play areas (eg, playground, fenced-in grass, and fenced-in pavement or rubber spaces; Figure 3). Programming follows more structured routines with educator-led activities, guided by the same provincial early learning framework that the experimental site follows [23]. Physical environments include standard play materials and fixed playground equipment. The 3 conventional ELCC centers are located in different settings, one in an urban area inside an apartment building and is licensed for 49 children (10 infants, 15 toddlers, and 24 preschoolers). One is located in an urban area in the basement of a church and is licensed for 39 children (15 toddlers and 24 preschoolers), and one is in a suburban neighborhood attached to an elementary school and is licensed for 31 children (15 toddlers and 16 preschoolers); this site also has a kindergarten and school-age program. All locations (including the experimental location) are part of the Canada-wide ELCC program, which in Ontario in 2026 means that the childcare fee is capped at CAD $22.00 (US $15.50) per day.

A priori power analysis was conducted using G*Power (Heinrich-Heine-Universität Düsseldorf) to estimate the sample size required to detect meaningful group differences over time for the primary objective of the study (ie, child outcomes). Given the complexity of general linear mixed-effects models and the limited availability of accessible power calculation tools for these models, a repeated-measures ANOVA with a within-between interaction was used as an approximation. This approach allows for estimating power to detect changes within participants over 3 time points and differences between 2 groups (experimental vs usual care). The calculation assumed a small effect size (*f*=0.20), a power of 0.80, and an alpha level of .05. This choice aligns with findings from systematic reviews indicating that ELCC interventions often yield small but statistically significant effects on children’s development [24]. Based on these parameters, the estimated required sample size is 42 child participants (21 per group). While our primary analyses use general linear mixed-effects models, this conservative ANOVA-based estimate provides a reasonable benchmark for planning purposes and may underestimate statistical power. Given the open cohort design, in which participants may enter and exit over time and contribute varying numbers of observations, precise specification of variance and correlation parameters is challenging at the design stage. In the absence of prior data, assumptions were therefore based on conservative estimates. To further strengthen these estimates, an internal pilot approach will be used whereby preliminary data from the first year of the study (ie, Wave 1: T0, T1, and T2) will be used to estimate key parameters (eg, variance components and within-subject correlation). These estimates will inform a refined assessment of sample size and statistical power for the remaining study period. This process will not involve formal hypothesis testing and will not affect the integrity of the primary analyses. The current sample size estimation is treated as a minimum threshold. In practice, we aim to enroll 100% of children attending the outdoor-focused ELCC center (ie, experimental site) and seek to match this number across the usual care sites. Therefore, we anticipate a substantially larger sample size that will improve the precision and generalizability of our findings [25].

### Data Collection Procedures

Data are collected by trained assessors following detailed standard operating procedures to ensure consistency across assessments. At the same time, a universal design approach [26] is taken to promote inclusion and accessibility for all children. For participants with identified disabilities, a member of the study team experienced in working with children with disabilities will consult with the classroom educator and the study team’s resource consultant in advance of the assessment to plan any necessary adaptations. While standardized procedures are followed whenever possible, assessments that a child does not have the ability to complete are not administered. In some cases, tasks may be modified to better align with a child’s abilities (eg, permitting use of a mobility aid when demonstrating a kicking task). All deviations from standard procedures are documented, and sensitivity analyses will be conducted to examine the impact of including or excluding children who did not complete the full standardized protocol on the study results.

**Figure 4.**
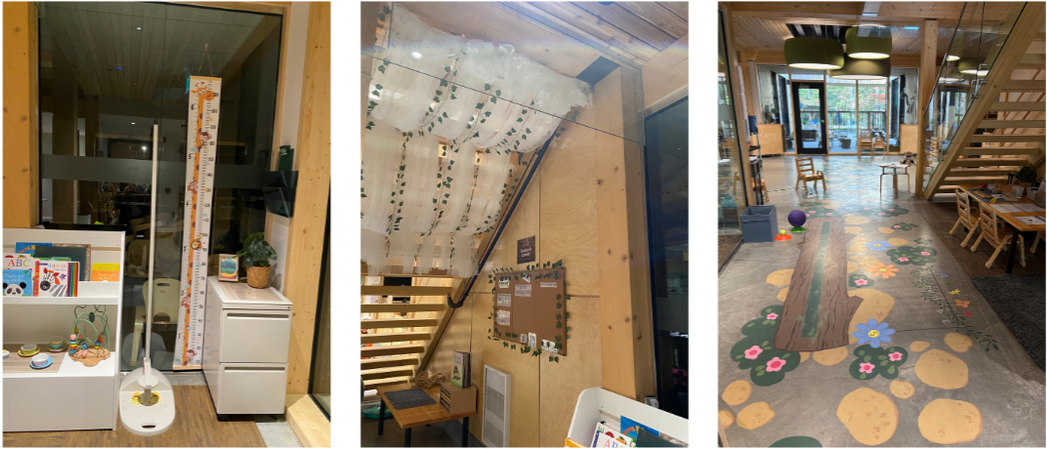
Research garden at the experimental Early Learning and Child Care program.

Assessments with children take place on-site during ELCC hours 3 times per year: baseline (T0), 6-month follow-up (T1), and 12-month follow-up (T2). For children who reenroll in the study after 1 year, assessments will continue every 6 months for up to a total of 6 follow-up assessments. There will be 3 waves of data collection representing 3 cohort groups across 3 years (Figure 3). Important to note, “waves” are distinct from time points; time points (eg, T0 and T1) refer to when data are collected during the study, whereas waves refer to when groups of participants are enrolled in the study. Testing of all children is typically completed over a 1-month period in the summer (August) and winter (February). To support children’s understanding of the research activities and to promote their engagement, a Research Garden (Figure 4) was created at the experimental ELCC program. This space offers a welcoming, familiar environment where children can explore materials, ask questions, and participate in tasks more comfortably. By embedding the research within an inviting, playful setting, the Research Garden helps motivate children, reduce apprehension, and foster a sense of curiosity and involvement in the study. Although we were unable to establish a Research Garden at usual care sites, we support children’s engagement through regular visits to the center to build familiarity with the research team. We also worked with the educators to select a temporary, quiet, child-friendly space for assessments and will use the same space for future assessments to build comfort. We also use engaging materials (eg, a teddy bear wearing an accelerometer) and small incentives (eg, stickers) to promote participation.

Caregiver questionnaires are distributed via REDCap, a secure, institutional web-based data management platform, following each assessment and are completed within 7 days of the assessment date. Each child is assigned a unique study ID and added to a password-protected master list to securely track data across all time points. During each assessment, children complete a battery of standardized assessments appropriate to their age group and ability (details provided below). Data are recorded on hard-copy data collection forms and then entered into REDCap following the assessment. One research staff member enters the data, and another verifies it for accuracy.

Educator questionnaires are distributed following the same schedule as child assessments. Educators receive a REDCap questionnaire 3 times per year and are asked to complete the questionnaire electronically within 7 days of receiving it. Educators are also invited to reenroll in the study for up to 3 years (or a maximum of 6 follow-up questionnaires).

### Outcome Measures

The objectives of this study will be assessed using a combination of direct child assessments and caregiver and educator questionnaires. These instruments are described in detail in the following section. A summary of measures for children, caregivers, and educators can be found in Table 1.

**Table 1.** Child, caregiver, and educator outcome variables and measures used in the SPROUT^a^ study.

Outcome and measure	Informant			
	Child	Caregiver	Educator	ELCC^b^ center
Child outcomes				
Physical development				
Motor skills				
PDMS-3^c^	✓			
Nine-Hole Peg Test	✓			
Supine Timed Up and Go	✓			
ASQ-3^d^		✓		
ECDI^e^		✓		
Upper limb strength				
Hand Grip Dynamometer	✓			
24-hour movement behaviors				
Accelerometer	✓			
Questionnaire		✓		
Social-emotional development				
Problem-solving				
ASQ-3		✓		
Social-emotional skills				
ASQ-3		✓		
ECDI		✓		
Cognitive development				
Executive functioning				
ECDI		✓		
Early Years Toolbox - Mr. Ant, Go/No-Go	✓			
Caregiver outcomes				
Affective and instrumental attitudes toward outdoor play				
Modified beliefs about caregiver support of child’s physical activity questionnaire		✓		
Outdoor play supports behaviors				
Modified caregiver support of child’s physical activity questionnaire		✓		
Educator outcomes				
Affective and instrumental attitudes toward outdoor play				
Modified beliefs about caregiveral support of child physical activity questionnaire			✓	
Outdoor play knowledge and confidence				
Early Childhood Educators’ Confidence in Outdoor Movement, Physical Activity, Sedentary and Screen behaviors (ECE-COMPASS) Questionnaire			✓	
Health and well-being				
Copenhagen Psychosocial Questionnaire, third edition (COPSOQ-III)			✓	
Other				
Injury statistics				
Author-generated questionnaire				✓
Quality of ELCC outdoor space				
Seven Cs Early Childhood Education Center assessment score				✓

a SPROUT: Sending Preschoolers Outside.

b ELCC: Early Learning and Child Care.

c PDMS-3: Peabody Developmental Motor Skills assessment, 3rd edition.

d ASQ-3: Ages and Stages Questionnaire, third edition.

e ECDI: Early Childhood Developmental Index.

### Child Assessments (Objective 1)

#### Overview

Child assessments are conducted individually onsite at the participating ELCC center and take approximately 45‐60 minutes per participant to complete. Assessments are conducted by researchers who have completed formal training on all assessments, including training on how to administer the different assessments among children with physical, sensory and/or developmental disabilities.

#### Anthropometry

For children aged 10‐24 months, body length is measured using an infant measuring rod (Seca GmbH & Co KG). Children are placed supine with their head aligned with the headboard and feet aligned with the footboard, straightening as best as possible. Weight is measured using a digital scale (Seca GmbH & Co KG) designed to measure child weight while in the arms of an assessor and measured to the nearest 0.1 kg. The assessor holds the child in their arms to complete the weight measurement, subtracting the assessor’s weight from the total to determine the child’s weight. For children aged 24‐59 months, the child’s height and weight are measured using a medical-grade digital scale (Seca GmbH & Co KG) and stadiometer (Seca GmbH & Co KG). If a child is 24‐59 months but has a disability that impacts their ability to stand prone without assistance (eg, cerebral palsy), the assessors will follow the standard procedures for children aged 10‐24 months (described above) that do not require a child to maintain an upright standing position without assistance, to measure height and weight.

For all children, regardless of age or disability, height and length and weight are calculated with the child’s shoes removed, and children are asked to remove any heavy clothing (eg, sweatshirt). Two measurements for height are taken to the nearest 0.1 cm. If measures differ by more than 0.5 cm, a third measurement is taken. The average of the 2 closest measurements is retained. Body weight is measured to the nearest 0.1 kg twice. Measurements are repeated a third time if they differ by more than 0.25 kg and an average of the 2 closest measurements is retained.

#### Physical Activity

Physical activity is measured using Actigraph accelerometers (Ametris, GT3XP-BTLE). The GT3XP-BTLE activity monitor is a small (3.8 cm × 3.7 cm×1.8 cm) light (27 g) sensor with no external controls or feedback. The accelerometer is secured by a belt and worn across the right hip. In line with existing recommendations for measuring children’s physical activity in the early years [27], children wear the accelerometer for 7 consecutive days (including during sleep), only removing it for prolonged water activities (eg, bathing and swimming). Caregivers are asked to keep a log of when the accelerometer is put on and taken off. To encourage compliance, the assessor explains to each child that the monitor is their “special belt” that measures their playing, resting, and sleeping, and the child is invited to choose a sticker to personalize their belt to take home. Physical activity data are analyzed using ActiLife software (version 6.15.0; ActiGraph) and R (version 4.4.2; R Core Team) [28] using RStudio version 2024.12.0+467 (Posit PBC) [29]. Data are downloaded in ActiLife using 15-second epoch lengths. Fifteen-second epoch lengths were chosen due to the short, sporadic activity bursts that are typical in young children [27]. Physical activity data are then processed and analyzed in RStudio. The *PhysicalActivity* package [30] is used to derive activity metrics from raw accelerometer data, including intensity classifications and summary variables. The *PhysActBedRest* package [31] supports the identification of sustained bedrest and waking periods to refine nonwear and rest interval detection. The *actigraph.sleepr* package [32] is used to score sleep and wake episodes based on ActiGraph accelerometer files, enabling the extraction of standardized sleep parameters. The Trost et al [33] and Pate et al [34] cut-points are used to determine minutes of light, moderate, and vigorous activity among toddlers (10‐35 months) and preschoolers (36‐48 months), respectively. The Tracy cut-points are used to determine hours slept [35]. If available, disability-specific cut-points will be used on a case-by-case basis for children with identified disabilities [36].

#### Motor Development

The Peabody Developmental Motor Skills assessment, 3rd edition (PDMS-3, Pearson Scientific) is used to measure children’s fine and gross motor skills. The PDMS-3 is designed to measure both fine and gross motor skills of children of various abilities from birth up to 6 years of age. The test is administered in-person by a trained researcher and takes between 30 and 45 minutes to complete depending on the child’s age and ability level. The PDMS-3 includes 5 core subtests, with 3 gross motor (body control, body transport, and object control) and 2 fine motor tests (hand manipulation and eye-hand coordination). A score is given based on the sum of each subtest. The PDMS-3 also produces a composite index score for gross and fine motor skills as well as a total score. The PDMS-3 has been previously established as a valid and reliable measure of motor skills in children with and those without disabilities aged 2‐4 years old [37].

The Nine-Hole Peg Test is used as a secondary measure of fine motor skills. The Nine-Hole Peg Test is completed by children aged 36 months and older in this study. The Nine-Hole Peg Test is part of the National Institutes of Health (NIH) toolbox [38] and is a widely used, reliable measure of manual dexterity in children with and those without disabilities [39,40]. The Nine-Hole Peg Test has also been shown to be a reliable measure of manual dexterity among children and youth with cerebral palsy [41].

The Supine Timed Up and Go (S-TUG) task is used as a secondary measure of gross motor skills, specifically a child’s motor coordination. The S-TUG task is completed by children aged 36 months and older in this study. S-TUG is considered a reliable measure of motor competence in children with high concurrent validity, interrater reliability, and intrarater reliability >0.80 [42]. Evidence also shows that the S-TUG tasks are highly reliable among children with cerebral palsy [43] and therefore by extension, may be accessible to children with similar physical disabilities.

A hand grip dynamometer is used to measure grip strength, an indicator of upper limb strength. The assessment is completed by children aged 36 months and older in this study. This assessment is also included as part of the NIH toolbox [38] and has been established as a valid, reliable, and feasible tool to measure upper limb strength in preschool-aged children, with an interclass correlation coefficient varying from 0.84 to 0.98 (intrarater) and from 0.81 to 0.96 (interrater).

#### Executive Functioning

Two tasks from the NIH Early Years Toolbox are used to assess visual-spatial working memory (Mr. Ant) and task inhibition (Go/No-Go). These tasks were developed as brief, playful assessments administered using an iPad to examine young children’s cognitive development. The Mr. Ant task assesses visual-spatial working memory. The Go/No-Go tasks assess a child’s inhibition. For more information on these assessments, see Early Years Toolbox [44]. These assessments have been previously validated in children aged 2.5‐5 years old and demonstrate very good reliability, convergent validity, and sensitivity [45,46]. Executive functioning tasks are completed by children aged 36 months and older in this study.

### Caregiver Questionnaire (Objective 1 and 2)

#### Overview

Caregivers complete an online questionnaire via REDCap (also made available in hard-copy format upon request) that has them report on family demographic characteristics, the child’s 24-hour movement behaviors (physical activity, sedentary behavior, and sleep), the home environment, aspects of the child’s development, and caregivers’ attitudes and behaviors toward active outdoor play. Each component of the questionnaire is briefly described below; for full details, refer to Multimedia Appendix 1.

#### Family Demographic Information

Demographic information of the caregiver and child collected includes caregiver age, sex, and gender; child age, sex, and gender; household income; and the outdoor environment at home.

#### 24-Hour Movement Behaviors and Outdoor Play

Items from the Canadian Health Measures Survey [47] are used to collect information on the child’s movement behaviors. Caregivers are also asked to report on the number of minutes their child spends outside playing on weekdays and weekend days.

#### Child Development

Child development is measured using 2 assessment tools completed by caregivers: the Early Childhood Developmental Index (ECDI) and the Ages and Stages Questionnaire, third edition (ASQ-3). The ECDI was developed by the United Nations Children’s Fund (UNICEF) to assess key child developmental milestones of children aged 24 to 59 months. The ECDI consists of 20 questions completed by a caregiver who asks them about the way their child behaves in different situations as well as their child’s skills and knowledge [48]. The ASQ-3 is a widely used screening tool for tracking and monitoring developmental progress among children aged 1 to 66 months that is completed by the caregiver [49]. The ASQ-3 measures development across 5 domains: communication, gross motor, fine motor, problem-solving, and personal-social, and has shown high validity and reliability [50].

#### Caregiver Attitudes and Behaviors Toward Outdoor Learning

Caregivers’ affective and instrumental attitudes and their current support behaviors toward outdoor play are measured using 6 items adapted from the caregiver support measure described by Rhodes et al [51] for school-aged children’s physical activity. Five items, also adapted from the caregiver support measure described by Rhodes et al [51], are used to measure caregivers’ current levels of support behaviors for outdoor play.

### Educator Questionnaire (Objective 2)

Each component of the educator questionnaire is briefly described below; for full details, refer to Multimedia Appendix 2.

#### Educator Health and Well-Being

Educator health and well-being are assessed using items from the Copenhagen Psychosocial Questionnaire, third edition. The Copenhagen Psychosocial Questionnaire, third edition, is an international research instrument designed for assessing and improving psychological conditions in the workplace [52]. International results showed acceptable to good reliability (Cronbach α >0.7) [52]. For the purpose of this study, we use items from the Job Satisfaction and Health and Wellbeing Scale.

#### Educator Knowledge and Confidence Toward Active and Outdoor Play

Educator knowledge and confidence toward outdoor play in ELCC are measured using the Early Childhood Educators’ Confidence in Outdoor Movement, Physical Activity, Sedentary and Screen behaviors Questionnaire. The Confidence in Outdoor Movement, Physical Activity, Sedentary and Screen Behaviors Questionnaire is a valid and reliable tool (test-retest statistics >0.62) that assesses educators’ task and barrier self-efficacy toward active outdoor play [53].

#### Educator Attitudes Toward Outdoor Play

Educators’ affective and instrumental attitudes toward active outdoor play are measured using items adapted from Rhodes et al [51] study exploring caregiver support for school-aged children’s physical activity. Six items are used to measure educators’ affective and instrumental attitudes toward supporting children’s outdoor play.

### ELCC Environment

#### Evaluation of the Outdoor Space

The Seven Cs framework [54] is used to assess the quality of the outdoor space at participating ELCC centers, using a checklist adapted for the following 7 criteria: character, context, connectivity, change, chance, clarity, and challenge. Two members of the research team visited each participating center to complete the checklist and achieve consensus on an overall quality score prior to the start of the study. The checklist took approximately 15 minutes to complete.

#### Injury Rates Within ELCC Programs

As part of this research study, we are also collecting child injury data to compare injury rates in outdoor-based versus conventional ELCC programs. These data are provided by participating ELCC programs and consist of a list of deidentified reported injuries (Multimedia Appendix 3).

### SPROUT-able Substudy (Objective 3)

As part of the SPROUT study, a substudy titled “SPROUT-able” is being conducted to assess social and built environmental barriers and facilitators to outdoor play and learning in natural spaces of the outdoor-focused ELCC program (experimental site) for children with disabilities and/or identified challenges in one or more functional domains (ie, seeing, hearing, walking, fine motor, communication, learning, and playing, behavior) [55].

### Community Engagement in SPROUT-able

To support meaningful community engagement in the SPROUT-able substudy, we formed a collaborative group of community partners and individuals invested in improving outdoor play opportunities for children with disabilities who have professional and/or lived experience with disability (which includes the children they work with, their own children, or themselves). These partners contribute lived and professional expertise to guide the substudy’s design and implementation. To clarify roles and support shared decision-making, we use the Involvement Matrix (Multimedia Appendix 4) [56]. The Involvement Matrix is a tool used in partner-engaged research to clarify and visualize the different roles and levels of participation that partners or stakeholders can take throughout the various phases of a research project. This approach promotes transparency, supports reciprocal learning, and ensures the research remains responsive to community priorities.

### Determining Disability Status

To identify children with a disability or who may potentially have a disability, we will use a combination of caregiver-reported diagnosis, a validated caregiver questionnaire of functional abilities, and reports from the inclusion support worker. As part of the SPROUT study caregiver questionnaire described previously, caregivers are asked whether their child has been diagnosed with a disability (yes, no, in the process of getting a diagnosis), and if yes, to provide a brief description. Recognizing that many disabilities go undiagnosed in early childhood [55], we have implemented 2 other processes for identifying children who may have additional challenges in one or more developmental domains that could impact their participation in outdoor play. First, all caregivers complete the Washington Group/UNICEF Child Functioning Modules - Ages 2‐4 Years [55]. This tool is designed to capture challenges a child experiences in daily functioning. Aligned with the International Classification of Functioning conceptual model of disability [57], this tool focuses on what children can do and the level of difficulty they experience, rather than on medical diagnoses. Questions cover 8 functional domains (ie, seeing, hearing, walking, fine motor, communication, learning, playing, and controlling behavior) with response options that capture different levels of difficulty to reflect the severity of a child’s functioning (eg, no difficulty, some difficulty, a lot of difficulty, and cannot do at all). For the purpose of the SPROUT-able study, children are identified as potentially having a disability if the caregiver indicates “a lot of difficulty” or “cannot do at all” in one or more functional domains, consistent with the Washington Group/UNICEF guidelines. Where appropriate, we will further categorize children by severity level (eg, low to high functional difficulty). Finally, the inclusion support worker, who works in collaboration with the study team and the ELCC center, will document which children require ongoing additional support in the classroom, indicating a potential disability or developmental challenge. The inclusion disability support worker will do this by completing a checklist that aligns with the Washington Group/UNICEF guidelines used in the caregiver questionnaire. Disability status is reassessed every 6 months, given the rapid developmental changes in early childhood and the evolving demands placed on children as they grow.

### Measuring Accessibility and Inclusion

#### GoPro Cameras

The use of GoPro cameras (GoPro Inc, HERO12 Black) captures visual and audio data of children’s perspectives and their unique play behaviors at the outdoor-focused ELCC program. GoPro cameras will be worn by children with and those without disabilities to compare play behaviors and physical activity participation. GoPros are placed on the child using the GoPro Junior Chesty (GoPro Inc, Junior Chest Mount Harness), which is designed for children ages 3‐14 years old weighing between 25 and 115 pounds. The use of chest-supported mounted GoPro cameras is deemed feasible in children aged 17‐25 months without impediment of balance due to additional weight [58]. Data collection occurs for approximately 1 to 2 hours during ELCC. Children are introduced to the GoPro cameras during a familiarization period prior to data collection. They are introduced to assessors who are present during times of collection and who invite them to wear a camera if they want to. During this familiarization period, all children are allowed to explore the GoPro cameras and chest straps to become familiar with the equipment. Throughout data collection, assessors and staff remain nearby in the event that children’s expressions or nonverbal cues suggest they are no longer comfortable wearing the GoPro cameras. Children are allowed to request to stop wearing the camera at any moment through any means of communication (eg, verbally, photo exchange communication). Audio and video collected from the GoPro cameras are uploaded to a secure institutional server accessible only to the research team at the end of each data collection session and are erased from GoPro storage. Given that there is a possibility of children and educators not participating in the study being captured on the GoPro footage, all caregivers of children and educators at the outdoor-focused ELCC will be notified about the research study. Only children who provide consent (ie, wear the GoPro) will be included in the analysis. Analyses will focus on each consenting child’s behavior independently, without directly coding or describing the behaviors of others (eg, noting that a child began playing following a positive or negative peer interaction, rather than detailing the peer’s specific actions).

All video and audio recordings will be deidentified (eg, names removed from audio transcripts). Video footage will be used for analysis purposes only, and no video footage will be shared publicly or beyond the research team. Once the video is analyzed and the study is complete, the video footage will be destroyed.

#### Educator, Caregiver, and Community Partner Feedback

To gather ongoing feedback on accessibility and inclusion as well as feasibility and acceptability at the outdoor-focused ELCC center, we collect annual qualitative data using World Café focus groups and individual interviews with caregivers and educators. The World Café method is a structured, participatory approach that facilitates collaborative dialogue through a series of small-group discussions focused on key questions [59]. Participants rotate between 3 to 5 tables, 20 minutes at a time, with each table addressing a specific aspect of accessibility and inclusion. Participants are invited to contribute using a variety of communication tools: speaking, writing on sticky notes, and molding with clay. A designated host remains at each table to summarize previous conversations and support continuity across groups. This method allows for the collection of diverse perspectives in an inclusive and conversational format. Insights generated through qualitative data collection will be synthesized and used to inform annual modifications to the program, ensuring it remains responsive to the needs of the community. World Café focus groups will be conducted once every 2 years.

In addition to World Café focus groups, semistructured one-on-one interviews will be conducted with caregivers and educators to explore the acceptability and feasibility of the outdoor-focused program, the barriers and facilitators to inclusive and accessible outdoor play, and their perceptions of the program’s impact on children’s development. The interviews will be guided by an interview guide that consists of 16 questions, each with a corresponding set of prompts. Within the interview guide, caregivers and educators will be prompted to discuss their perspectives on the children’s experiences at the outdoor-focused program and their views on children’s physical, cognitive, and social-emotional development since beginning the program. Caregivers and educators will also be prompted to discuss the barriers and facilitators to outdoor play that children with and those without disabilities face in the outdoor-focused program. Each interview will take approximately 45‐60 minutes to complete and will be conducted on Zoom (Zoom Communications, Inc). The interviews will be audio and video recorded; however, only audio files will be retained for study purposes. Interviews will be conducted yearly and will prioritize caregivers of children in the SPROUT-able substudy.

### Data Analysis Plan: Primary Objective and Secondary Objectives 1 and 2

Descriptive statistics are used to summarize participant characteristics and all primary measures, including developmental assessments, movement behaviors, caregiver attitudes, support behaviors, and educator outcomes. Means and SDs are reported for continuous variables, and frequencies and percentages for categorical variables. General linear mixed-effects models will be used for the primary objective and secondary objectives 1 and 2 to examine differences in change over time and between groups. Separate models will be specified for child developmental outcomes (eg, motor, cognitive, and social-emotional), movement behaviors (eg, physical activity, sedentary time, and sleep), caregiver attitude and support measures, and educator-reported measures (eg, job satisfaction, health and well-being, and attitudes toward outdoor play). Models will include fixed effects for time (eg, T0 and T1), group (experimental vs usual care), and relevant covariates (eg, child age, sex, disability status, race and ethnicity, parent income, parent education, and newcomer status). For covariates with small subgroup sizes, distributions will be examined prior to modeling; where sparse categories are identified, categories will be collapsed as appropriate to ensure stable estimation. A random effect for participant (study ID) will be included to account for within-subject correlations across time points. The primary analyses will treat the 3 usual care sites as a single group. Sensitivity analyses will additionally adjust for the ELCC site (included as a fixed effect) to examine potential differences between individual usual care centers and to evaluate the extent to which such differences may influence the results.

Interaction terms (eg, group × time) are tested to examine whether changes over time differ between the experimental and usual care group, thereby estimating intervention effects as differences in change from baseline. Because baseline measurements are included as part of the repeated outcome in the mixed-effects models, this approach inherently adjusts for baseline differences between groups. The mixed-effects models accommodate unbalanced data, such that participants are not required to have observations at all time points; individuals with missing baseline or follow-up measurements contribute all available data to the analysis. These missing time points are handled within the maximum likelihood framework under a missing at random assumption, avoiding listwise deletion. Missing data within completed measurement occasions (eg, partial or item-level missingness) will be explored and, where appropriate, addressed using additional methods (eg, multiple imputation) based on the nature and extent of missingness. Model assumptions are checked using residual diagnostics. Statistical significance is set at less than *P*=.05. Effect sizes are computed and interpreted using established guidelines, with CIs reported to aid interpretation of both statistical and practical significance. Interim analyses are conducted after each year of the study and final analyses will be conducted after all data collection is complete (Figure 1). All analyses are conducted in R version 4.4.2 [28] using RStudio version 2024.12.0+467 [29].

### Secondary Objective 3

#### Go-Pro Data

GoPro data will be analyzed qualitatively to capture activity participation and barriers and facilitators to outdoor play and quantitatively to analyze the level of physical activity the child engages in. Our qualitative approach to analyzing the GoPro data is in line with a previous study examining outdoor play in young children using GoPro devices [58]. The GoPro’s recorded audio and video information will be transcribed by the research team and analyzed using NVivo12 software (QSR International). Using an inductive thematic analysis, this method will allow for similarities and differences throughout the data set to be highlighted and later triangulated using both audio and visual data from the GoPro video [60]. The approach to inductive thematic analysis will follow Braun and Clarke’s recommendations [61]. First, researchers will individually transcribe the videos. We will use a combination of verbatim transcription and visual transcription to capture both audio and visual information from the GoPro videos [62]. Next, the researchers will generate initial codes. To do this, transcriptions will be synced to the original media file to allow the coders to have access to the richness of the original video, along with the transcribed data. This approach is recommended when analyzing video data [62]. After the initial coding stage, researchers will combine all codes into one document and discuss themes that emerge from the coding. When reviewing the identified themes, video recordings will be used to further enhance the themes by comparing the nuances of video data to the themes found in transcripts. We will use a modified version of the Observational System for Recording Activity in Children – Developmental Delay, an observation tool validated for children with disabilities in the early years to quantify physical activity levels in the GoPro videos [63]. Using momentary time sampling, data will be coded for the first 5 seconds of every 30 seconds [15]. This will allow for a systematic observation of child activity which is more replicable across observers.

#### World Café Focus Groups and Interviews

An inductive thematic analysis will be used to identify key themes from the World Café focus groups and individual interviews [61]. The analysis will follow the 6 steps of thematic analysis described in detail above [61]. Pseudonyms will be used for all participant names in order to ensure confidentiality. Themes will be generated under three categories: (1) acceptability and feasibility of the outdoor-focused program; (2) perceived changes in children’s physical, cognitive, and social-emotional development; and (3) barriers and facilitators to supporting outdoor play for children with and those without disabilities.

## Results

**Figure 5.**
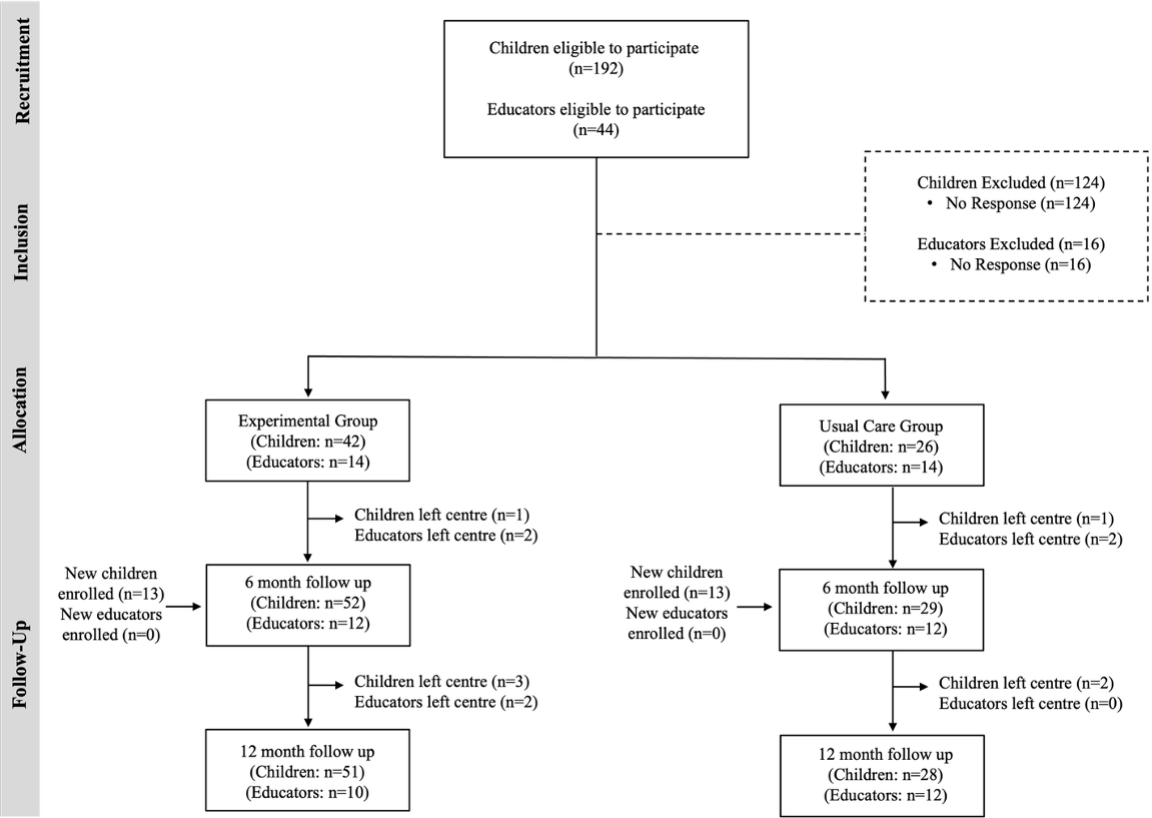
CONSORT (Consolidated Standards of Reporting Trials) flow diagram of participants in Wave 1.

Funding for the SPROUT and SPROUT-able studies was obtained in November 2022 and January 2024, respectively. Ethics approval from the Institutional Research Ethics Board was obtained in April 2024, and data collection began in July 2024, approximately 2 weeks after the experimental site opened. The study has currently completed 1 out of 3 years (waves) of data collection. A total of 110 children and their caregivers are currently enrolled; 67 are from the experimental site and 43 from the usual care sites. Sixty-two children who were enrolled at the beginning of the study (August 2024) have completed their baseline, T1, and T2 assessments (39 experimental and 23 usual care). Eighteen children who were enrolled in February 2025 (late enrollment, missing a baseline assessment) have completed their T1 and T2 assessments (13 experimental and 5 usual care). These children comprise Wave 1 of the study cohort. As of August 2025, 19 children attending the experimental site met the criteria for being in the SPROUT-able study. Of those children, 4 had a formal diagnosis of disability, 3 had caregiver-reported functional difficulties, and 12 children were receiving additional support from an inclusion support worker. At baseline, 28 educators enrolled in the study (14 experimental and 14 usual care; Wave 1). Of those educators, 6 (4 experimental and 2 usual care) left the center between T0 and T2. Figure 5 presents a breakdown of participants from Wave 1 of the study. For a complete description of the time-invariant variables of this study sample, refer to Table 2.

**Table 2.** Demographic characteristics of the study sample in Wave 1 at baseline as of February 2025.

Characteristics	All	Experimental	Usual care
Caregiver-child dyad demographic characteristics			
Age (months), mean (SD)	29.8 (10.9)	28.4 (12.0)	32.4 (6.9)
Child’s sex, n/N (%)			
Male	41/86 (47.7)	27/54 (50.0)	5/32 (15.6)
Female	45/86 (52.3)	27/54 (50.0)	17/32 (53.1)
Intersex	0/86 (0)	0/54 (0)	0/32 (0)
Child experiences disability, n/N (%)			
Yes	5/86 (5.8)	3/54 (5.6)	2/32 (6.3)
No	78/86 (90.7)	48/54 (88.9)	21/32 (65.6)
Unsure	3/86 (3.5)	3/54 (5.6)	0/32 (0)
Prefer not to answer	0/86 (0)	0/54 (0)	0/32 (0)
Child’s race, n/N (%)			
Black	1/86 (1.2)	3/54 (4.6)	1/32 (2.8)
East Asian	3/86 (3.5)	3/54 (4.6)	4/32 (11.1)
Indigenous	0/86 (0)	0/54 (0)	1/32 (2.8)
Latin American	3/86 (3.5)	3/54 (4.6)	1/32 (2.8)
Middle Eastern	3/86 (3.5)	4/54 (6.2)	3/32 (8.3)
South Asian	2/86 (2.3)	1/54 (1.5)	3/32 (8.3)
South East Asian	0/86 (0)	0/54 (0)	0/32 (0)
White	57/86 (66.3)	50/54 (76.9)	21/32 (58.3)
Multiple and mixed race	15/86 (17.4)	11/54 (20.4)	4/32 (12.5)
Prefer not to answer	2/86 (2.0)	0/54 (0)	2/32 (5.6)
Relationship to child, n/N (%)			
Mother	74/86 (86.0)	47/54 (87.0)	27/32 (84.4)
Father	12/86 (14.0)	7/54 (13.0)	5/32 (15.6)
Grandmother	0/86 (0)	0/54 (0)	0/32 (0)
Grandfather	0/86 (0)	0/54 (0)	0/32 (0)
Legal guardian	0/86 (0)	0/54 (0)	0/32 (0)
Other	0/86 (0)	0/54 (0)	0/32 (0)
Prefer not to answer	0/86 (0)	0/54 (0)	0/32 (0)
Partner’s education level, n/N (%)			
No formal schooling	0/86 (0)	0/54 (0)	0/32 (0)
Primary school	0/86 (0)	0/54 (0)	0/32 (0)
Secondary or high school	9/86 (10.5)	6/54 (11.1)	3/32 (9.4)
Vocational and college education	20/86 (23.3)	16/54 (29.6)	4/32 (12.5)
Tertiary and university education	54/86 (62.8)	31/54 (57.4)	23/32 (71.9)
Prefer not to answer	0/86 (0)	0/54 (0)	0/32 (0)
Not applicable	3/86 (3.5)	1/54 (1.9)	2/32 (6.3)
Caregiver or caregiver education level, n/N (%)			
No formal schooling	0/86 (0)	0/54 (0)	0/32 (0)
Primary school	0/86 (0)	0/54 (0)	0/32 (0)
Secondary or high school	2/86 (2.3)	1/54 (1.9)	1/32 (3.1)
Vocational and college education	13/86 (15.1)	11/54 (20.4)	2/32 (9.4)
Tertiary and university education	71/86 (82.6)	42/54 (77.8)	29/32 (87.5)
Prefer not to answer	0/86 (0)	0/54 (0)	0/32 (0)
Not applicable	0/86 (0)	0/54 (0)	0/32 (0)
Caregiver or caregiver’s birthplace, n/N (%)			
Both caregivers born in Canada	56/86 (65.1)	37/54 (68.5)	19/32 (59.4)
At least one caregiver born outside of Canada and moved to Canada <5 years ago	5/86 (5.8)	1/54 (1.9)	4/32 (12.5)
At least one caregiver born outside of Canada and moved to Canada, and moved to Canada 5 to 10 years ago	9/86 (10.5)	6/54 (11.1)	3/32 (9.4)
At least one caregiver born outside of Canada, and moved to Canada >10 years ago	15/86 (17.4)	10/54 (18.5)	5/32 (15.6)
Prefer not to answer	1/86 (1.2)	0/54 (0)	1/32 (3.1)
Educator demographic characteristics			
Age (years), mean (SD)	38.6 (11.6)	34.6 (8.9)	42.8 (12.9)
Sex, n/N (%)			
Female	26/27 (96.3)	14/14 (100)	12/13 (92.3)
Male	1/27 (3.7)	0/14 (0)	1/13 (7.7)
Intersex	0/27 (0)	0/14 (0)	0/13 (0)
Unknown	0/27 (0)	0/14 (0)	0/13 (0)
Gender, n/N (%)			
Women	25/27 (92.6)	13/14 (92.9)	12/13 (92.3)
Men	1/27 (3.7)	0/14 (0)	1/13 (7.7)
Another gender	0/27 (0)	0/14 (0)	0/13 (0)
Unknown	0/27 (0)	0/14 (0)	0/13 (0)
Not applicable	1/27 (3.7)	1/14 (7.1)	0/13 (0)
Race, n/N (%)			
Black	2/27 (8.0)	0/14 (0)	2/13 (18.2)
East Asian	3/27 (12.0)	1/14 (7.1)	2/13 (18.2)
Indigenous	0/27 (0)	0/14 (0)	0/13 (0)
Latin American	1/27 (4.0)	1/14 (7.1)	0/13 (0)
Middle Eastern	1/27 (4.0)	1/14 (7.1)	0/13 (0)
South Asian	1/27 (4.0)	0/14 (0)	1/13 (9.1)
South East Asian	0/27 (0)	0/14 (0)	0/13 (0)
White	16/27 (64.0)	11/14 (78.6)	5/13 (45.5)
Multiple and mixed race	0/27 (0)	0/14 (0)	0/13 (0)
Other	1/27 (4.0)	0/14 (7.1)	1/13 (9.1)

## Discussion

### Study Significance

The SPROUT study is the first in Canada, and to our knowledge globally, to directly and longitudinally compare a licensed outdoor-focused ELCC program with conventional ELCC programs. This research addresses a significant gap in the current evidence base regarding the short- and long-term developmental and health impacts of outdoor-focused ELCC models. By using a robust and appropriately powered design, the SPROUT study provides a unique opportunity to examine whether and how outdoor-focused ELCC models influence not only child health, development, and well-being, but also caregiver beliefs and attitudes, and the well-being, job satisfaction, and continued professional development of educators.

Importantly, the SPROUT study explores how outdoor-focused programs affect educators’ knowledge and attitudes toward outdoor play. These insights may support strategies to retain and strengthen the early childhood education workforce, a major challenge and need in Canada, given the new Canada-wide Early Learning and Child Care Act, which commits to adding 250,000 new licensed ELCC spaces in Canada by March 2026 [64]. The study also examines caregiver attitudes and support behaviors toward outdoor play, offering valuable information on how these beliefs evolve over time and differ across program types. This knowledge will serve to expand on the growing body of work being done in Canada exploring caregiver and educator perspectives of outdoor play and learning and how to support change toward promoting more outdoor play opportunities for all children [11,65]. Together, this body of literature may help inform strategies to promote outdoor play in ELCC, at home, and in community spaces.

The SPROUT study and the SPROUT-able substudy place a strong emphasis on inclusion by intentionally examining outcomes among children with disabilities. Children with disabilities are frequently excluded from ELCC settings or experience marginalization while enrolled [66]. Exclusion especially occurs during outdoor play, where both physical and social barriers are common [13-16]. By investigating how outdoor and nature-focused programs can foster environments that support the full participation of children with and those without disabilities, this study will provide insight into how outdoor play can potentially be used as a strategy to promote inclusion. In addition, by intentionally including children with disabilities, this study will be able to glean insight into the potential benefits of outdoor play and learning on child health and development for children with disabilities, an area of research recently identified as requiring more robust experimental evidence [67]. With these findings, there may be an opportunity to inform not only licensed ELCC practices but also broader community-based outdoor play initiatives and early years programs aimed at reducing barriers and increasing access to play outdoors and in nature for children with disabilities.

### Strengths and Limitations

This study has several notable strengths. The longitudinal cohort design enables temporal comparisons between outdoor-focused and conventional ELCC programs, providing insight into developmental changes over time. The study is appropriately powered, and the use of a naturalistic cohort design adds rigor and ecological validity. A comprehensive set of valid and reliable child outcome assessments, including both direct measures and caregiver-reported tools, ensures robust data collection. Additionally, this study examines changes in educator well-being, an area that has received little attention in outdoor-focused ELCC programs.

Despite this study’s strengths, there are limitations that we anticipate having a potential impact on the findings. Due to the naturalistic design of this study, randomization is not possible, which may influence causal inferences. Families self-enroll in the outdoor-focused program, introducing the potential for selection bias. However, both the experimental and usual care sites are funded through the Canada-wide Early Learning and Childcare program, where caregivers put their name on a city-wide waitlist and typically accept whatever program has capacity. Similarly, educators also self-select and apply to work at the center of their choice, and the outdoor-focused ELCC prioritizes educators who have training in outdoor-focused childcare, introducing a possible selection bias. To address this, we will explore baseline differences and focus our analyses on differences in change over time, inherently accounting for individual differences. Accessibility is also limited, as the outdoor-focused center is located outside the city center and is not yet reachable by public transit, potentially leading to a less diverse sample. Selection bias is likely, as families participating in the SPROUT and/or SPROUT-able studies may have an interest in child development, movement behaviors, and/or outdoor play, which could influence study outcomes. Finally, while a strength of this study is the intentional inclusion of children with disabilities, the study remains limited by a lack of valid and reliable measures of child development and health for diverse disability types.

### Conclusion

By taking a holistic and comparative approach that includes children with and those without disabilities, caregivers, and educators, the SPROUT and SPROUT-able studies offer comprehensive evidence to inform early childhood research and ELCC practice and policy in Canada and internationally. As countries look for sustainable, inclusive, and evidence-informed system approaches to early learning and development, this study provides a timely and evidence-based Canadian model that can guide efforts to better integrate inclusive outdoor play into early learning systems. Ultimately, this study has the potential to drive meaningful and inclusive improvements in ELCC settings by demonstrating if and how outdoor-focused programs can promote child development and healthier communities.

## Supplementary material

10.2196/89405 Multimedia Appendix 1 Parent questionnaire.

10.2196/89405 Multimedia Appendix 2 Educator questionnaire.

10.2196/89405 Multimedia Appendix 3 Injury statistics questionnaire.

10.2196/89405 Multimedia Appendix 4 SPROUT-able Involvement Matrix.

## Abbreviations

ASQ-3: Ages and Stages Questionnaire, third edition
ECDI: Early Childhood Developmental Index
ELCC: Early Learning and Child Care
NIH: National Institutes of Health
PDMS-3: Peabody Developmental Motor Skills assessment 3rd edition
S-TUG: Supine Timed Up and Go
SPROUT: Sending Preschoolers Outside
UNICEF: United Nations Children's Fund
