# Supplementary material for: Effect of an Outdoor-Focused Licensed Child Care Program on Child, Caregiver, and Educator Outcomes, Inclusion, and Accessibility: Protocol for the Sending Preschoolers Outside (SPROUT) Prospective Cohort Study
Source: JMIR Res Protoc. 2026 Jul 21;15:e89405. doi: 10.2196/89405 (PMC13387637; doi:10.2196/89405)
Supplement: Multimedia Appendix 1 [file resprot-v15-e89405-s001.docx]

**Appendix A: Parent Questionnaire**

| **Part 1. Background information about you and your child** | |
| --- | --- |
| **1.** | What is the date of birth of your child? (mm/yyyy) |
|  | __________/______________ |
| **2.** | What is your relationship to the child participating in the study? |
|  | Mother  Father  Grandmother  Grandfather  Legal Guardian  Other (please specify): _______________________________ |
| **3.** | What is the sex of your child? |
|  | - Female - Male - Intersex - Unknown - Prefer not to answer |
| **4.** | Does your child experience a disability? |
|  | - Yes - No - Unsure   Describe:   - Prefer not to answer |
| **5.** | If parent answered ‘yes’ to question #4:  What type of disability does your child experience? |
|  | - Developmental (e.g., Autism Spectrum Disorder, Down syndrome)   Describe:   - Physical (e.g., Cerebral palsy)   Describe:   - Sensory (e.g., blindness/low vision, sensory processing disorder)   Describe:   - Other   Describe:   - Multiple disabilities   Describe:   - I am not sure - Prefer not to answer |
| **6.** | If parent answered ‘yes’ to question #4:  Has your child received a diagnosis for this disability from a medical professional? |
|  | - Yes - No - In the process of getting a diagnosis - Prefer not to answer |
| The following questions ask about difficulties your child may have doing certain activities. When answering the questions, please think about your child in comparison to other children their age. | |
| **7.** | Does your child wear glasses or contact lenses? (Skip to question 9 if “no”)  Yes  No |
| **8.** | When your child wears their glasses or contact lenses, do they have difficult seeing?   - No difficulty - Some difficulty - A lot of difficulty - Cannot see at all - Prefer not to answer   Skip to question 10 |
| **9.** | Does your child have difficulty seeing? |
|  | - No difficulty - Some difficulty - A lot of difficulty - Cannot see at all - Prefer not to answer |
| **10.** | Does your child use a hearing aid? (Skip to question 12 if no) |
|  | - Yes - No |
| **11.** | When using their hearing aid, does your child have difficulty hearing sounds like peoples’ voices or music? |
|  | - No difficulty - Some difficulty - A lot of difficulty - Cannot see at all - Prefer not to answer |
| **12.** | Does your child have difficulty hearing sounds like peoples’ voices or music? |
|  | - No difficulty - Some difficulty - A lot of difficulty - Cannot hear at all - Prefer not to answer |
| **13.** | Does your child use any equipment or receive assistance for walking? If no skip to question 16  Yes  No |
| **14.** | Without their equipment or assistance, does your child have difficulty walking? |
|  | - No difficulty - Some difficulty - A lot of difficulty - Cannot do it at all - Prefer not to answer |
| **15.** | With their equipment or assistance, does your child have difficulty walking? |
|  | - No difficulty - Some difficulty - A lot of difficulty - Cannot do it at all - Prefer not to answer |
| **16.** | Compared with children of the same age, does your child have difficulty walking? |
|  | - No difficulty - Some difficulty - A lot of difficulty - Cannot do it at all - Prefer not to answer |
| **17.** | Compared with children of the same age, does your child have difficulty picking up small objects with their hand? |
|  | - No difficulty - Some difficulty - A lot of difficulty - Cannot do it at all - Prefer not to answer |
| **18.** | Does your child have difficulty understanding you? |
|  | - No difficulty - Some difficulty - A lot of difficulty - Cannot do at all - Prefer not to answer |
| **19.** | When your child speaks, do you have difficulty understanding them? |
|  | - No difficulty - Some difficulty - A lot of difficulty - Cannot do it at all - Prefer not to answer |
| **20.** | Compared with children of the same age, does your child have difficulty learning things? |
|  | - No difficulty - Some difficulty - A lot of difficulty - Cannot do at all - Prefer not to answer |
| **21.** | Compared with children of the same age, does your child have difficulty playing? |
|  | - No difficulty - Some difficulty - A lot of difficulty - Cannot do at all - Prefer not to answer |
| **22.** | Compared with children of the same age, how much does your child kick, bite or hit other children or adults? |
|  | - Not at all - The same or less - More - A lot more - Prefer not to answer |
|  |  |
| **23.** | What is your postal code? |
|  | _ _ _ _ _ _ |
| **24.** | How would you best describe the outdoor space where you and your child most often go for leisure and play? |
|  | - Private backyard - Shared backyard - Public park - Street - Alleyway - We do not spend time outdoors - Other (please describe): _________________________ |
| **25.** | **How would you rate the overall atmosphere (character, softness) of the outdoor space you most often go to with your child for leisure and play?** |
|  | - Very poor - Poor - Neutral - Good - Very good |
| **26.** | **How would you rate the health and safety (secure from dangers, vandalism, animal invasion) of the outdoor  space you most often go to with your child for leisure and play?** |
|  | - Very poor - Poor - Neutral - Good - Very good |
| **27.** | What is your (parent/caregiver) age in years? |
|  | __________ |
| **28.** | What was your sex at birth? |
|  | - Female - Male - Intersex - Unknown - Prefer not to answer |
| **29.** | What is your current gender identity? |
|  | - Female - Male - Another Gender   What gender do you identify as? ___________________   - Unknown - Not applicable - Prefer not to answer |
| **30.** | What is the highest level of education you have completed? |
|  | - No formal schooling - Primary school - Secondary or high school - Vocational/College education - Tertiary/University education - Prefer not to answer - Not applicable |
| **31.** | What is the highest level of education your partner (if applicable) has completed? |
|  | - No formal schooling - Primary school - Secondary or high school - Vocational/College education - Tertiary/University education - Prefer not to answer - Not applicable |
| **32a.** | In our society, people are often described by their race or racial background. These are not based in science, but our race may influence the way we are treated by individuals and institutions, and this may affect our health. Which category(ies) best describes your child? Check all that apply: |
|  | Black (e.g., African, African Canadian, Afro-Caribbean descent)  East Asian (e.g., Chinese, Japanese, Korean, Taiwanese descent)  Indigenous (e.g., First Nations, Inuk/Inuit, Métis descent)  Latin American (e.g., Hispanic or Latin American descent)  Middle Eastern (e.g., Arab, Persian, West Asian [e.g., Afghan, Egyptian, Iranian, Kurdish, Lebanese, Turkish] descent)  South Asian (e.g., Bangladeshi, Indian, Indo-Caribbean, Pakistani, Sri Lankan descent)  Southeast Asian (e.g., Cambodian, Filipino, Indonesian, Thai, Vietnamese descent)  White (e.g., European descent)  Do not know  Prefer not to answer  Another race category (Optional - please specify:) _______________________________ |
| **32b.** | Where were you and your partner (if applicable) born? |
|  | Both parents born in Canada  At least one parent born outside of Canada, and moved to Canada less than 5 years ago  At least one parent born outside of Canada, and moved to Canada 5 to 10 years ago  At least one parent born outside of Canada, and moved to Canada more than 10 years ago  Do not know  Prefer not to answer |

**[Items from the ASQ will be inserted here]**

| **Part 2: Information about your child’s movement behaviours** | | | | |
| --- | --- | --- | --- | --- |
| The next questions ask about your child's movement behaviours. Please report the **number of hours and minutes per day** (for all the questions). E.g. 1 hour and 30 mins | | | | |
| **33.** | In the past week, were there any days where your child in this study was restrained for more than one hour at a time in a stroller, car seat, high chair? | | | |
|  | Yes  No | | | |
| **34.** | Over a **typical** **weekday**, how much time does your child spend as a passenger in a motor vehicle (e.g. a car, bus) | | ………….hrs ………………..mins | |
| **35.** | Over a **typical** **weekend day**, how much time does your child spend as a passenger in a motor vehicle (e.g., a car, bus, motorcycle) | | ………….hrs ………………..mins | |
| **36.** | On a typical **weekday**, how much time does your child spend outside? | | ………….hrs ………………..mins | |
| **37.** | On a typical **weekend day**, how much time does your child spend outside? | | ………….hrs ………………..mins | |
| **38a.** | In the past three days, has your child: Gone outside the home to play (alone/with an adult/older child)? | | | |
|  | Yes  No | | | |
| **38b.** | **If Yes**, where did the child go? **(tick all that apply?)**  On the property (i.e. Garden/yard)  To a friend/relative’s home  To a park/square or playground  To a swimming pool/creek/river/dam/waterhole/beach  To the street  To the forest/nature environment  Other? (please specify)  ______________________________________________________________ | | | |
| **38c.** | In the past three days, has your child: **NOT** gone/been allowed to go/taken outside to play because of **(check all that apply)** | | | |
|  | Heat  Noise or crowding  Cold  Garbage/rubbish  Rain  Social unrest  Air pollution (dirty, smoky, smelly)  Dangers such as crime/kidnapping  Water pollution  Dangers such as traffic  Other? (please specify)  ……………………………………………………………………………………………………………………….  Not relevant | | | |
| **39.** | **In a 24-hour period in the past week**, how much time did your child spend using any electronic screen device such as a smart phone, tablet, video game, or watch television or movies, videos on the internet while they were sitting or lying down? Please record this as accurately as you can. | | | ………….hrs  …………..mins |
| **40.** | How often do you use an electronic screen device to educate your child? | | | |
|  | Never  Less than once a week  Once a week  Most days  Every day  Don’t know  Prefer not to answer | | | |
| **41.** | How often do you use an electronic screen device to calm down your child when they are upset? | | | |
|  | Never  Less than once a week  Once a week  Most days  Every day  Don’t know  Prefer not to answer | | | |
| **42.** | How often do you use an electronic screen device to keep your child busy while you get things done? | | | |
|  | Never  Less than once a week  Once a week  Most days  Every day  Don’t know  Prefer not to answer | | | |
| **43.** | How often do you use a smartphone to make calls, text messages, check email, check social media, watch a video during meals with your child? | | | |
|  | Never  Less than once a week  Once a week  Most days  Every day  Don’t know  Prefer not to answer | | | |
| **44.** | How often do you use a smartphone to make calls, text messages, check email, check social media, watch a video during playtime with your child? | | | |
|  | Never  Less than once a week  Once a week  Most days  Every day  Don’t know  Prefer not to answer | | | |
| **45.** | How often do you use a smartphone to make calls, text messages, check email, check social media, watch a video during travel time with your child? | | | |
|  | Never  Less than once a week  Once a week  Most days  Every day  Don’t know  Prefer not to answer | | | |
| **46.** | How often do you use a smartphone to make calls, text messages, check email, check social media, watch a video while going for walks with your child? | | | |
|  | Never  Less than once a week  Once a week  Most days  Every day  Don’t know  Prefer not to answer | | | |
| **47.** | How often do you use a smartphone to make calls, text messages, check email, check social media, watch a video during bedtime routine with your child? | | | |
|  | Never  Less than once a week  Once a week  Most days  Every day  Don’t know  Prefer not to answer | | | |
| **48a.** | Does your child use electronic screen devices (e.g. TV, video game, computer, tablet or smartphone) in the 2 hours before bedtime on a daily basis? **If no, go to question 28** | | | |
|  | Yes  No  Don’t know  Prefer not to answer | | | |
| **48b.** | **If Yes**, how close to bedtime does your child usually use these devices? | | | |
|  | Closer than 30 minutes before bedtime  30 mins to less than 1 hour before bedtime  Between 1 and 2 hours before bedtime  Prefer not to answer | | | |
| **49.** | Does your child have electronic screen devices in the room where they sleep (e.g. TV, video game, computer, tablet or smartphone)? | | | |
|  | Yes  No  Prefer not to answer | | | |
| **50a.** | Please indicate your agreement with the following statement: Thinking about the last 6 months, my child’s screen media habits are problematic (e.g., hard to stop using, amount of time keeps increasing, frustrated or unmotivated when unable to use screen media)? | | | |
|  | o Strongly disagree  o Disagree  o Neither disagree nor agree  o Agree  o Strongly agree  o Don’t know  o Not applicable  Prefer not to answer | | | |
| **50b.** | At what age did your child start using any screens? | | | |
|  | 🞏 0-12 months  🞏 13-18 months  🞏 Over 18 months  🞏 They don’t use screen media  Prefer not to answer | | | |
| **51.** | How many hours of sleep does your child get in a typical 24-hours day (including naps)? | | ………….hrs ………………..mins | |
| **52a.** | Does your child nap? **If yes, go to 42b** | **52b.** | **If yes**, what time does your child typically nap? | |
|  | Yes  No | Begin time: …………………….  End time: ……………………… | | |
| **53a.** | Does your child have a consistent bedtime? | **53b.** | Does your child have a consistent wake-up time? | |
|  | Yes, bedtime does not vary by more than 30 minutes each day  No, bedtime can vary more than 30 minutes each day |  | Yes, wake-up time does not vary by more than 30 minutes each day  No, wake-up time can vary more than 30 minutes each day | |
| **54a.** | What time does your child go to bed at night? | **54b.** | What time does your child get up in the morning? | |
|  | Bed time: ……………………. PM | Wake-up time: ……………………… AM | | |
| **55.** | On a scale of 1 to 7, with the higher number indicating higher quality, how would you rate the quality of your child's sleep?  1 would indicate very difficult to settle, wakes many times during the night for prolonged periods and is very restless (tosses and turns, throw off bedclothes) while 7 would indicate settles and drifts off to sleep for a few minutes, sleeps right through the night, and has a very sound, deep sleep) | | | |
|  | 1  2  3  4  5  6  7  Don’t know  Prefer not to answer | | | |
| **56a.** | In the past three days, has your child: **NOT** gotten enough sleep? | | | |
|  | Yes  No | | | |
| **56b.** | **If Yes**, was it because of: **(tick all that apply?)** | | | |
|  | Outside noise (like traffic/train/street noises)  Indoor noise  Too Hot  Too cold  Too much light coming in to the room  Other (please specify)………………………………………………………………………………………………………………………. | | | |
| **57a** | Does your child sleep in their own bedroom by themselves? | | | |
|  | Yes  No | | | |
| **57b.** | **If No,** how many other people sleep in the same room as your child? | | | |
|  | …………………Children …………………. Adults (18 years and older) | | | |
| **57c.** | **If No,** how many other people sleep in the same bed as your child? | | | |
|  | ………………None …………………Children …………………. Adults (18 years and older) | | | |
| **58.** | How often is there a bedtime routine for your child (e.g., bath time, saying goodnight, storytelling, etc)? | | | |
|  | Never  Less than once a week  Once a week  Most days  Every day  Don’t know | | | |
| **Part 3. Information about your support for outdoor active play** | | | | |
|  | The following questions are going to ask about your support with regard to your child’s **active outdoor play.** | | | |
| **59.** | \|  \|  \|  \|  \|  \|  \| \| --- \| --- \| --- \| --- \| --- \| --- \| \|  \| **Never/Rarely** \| **1 to 2 times per week** \| **3 to 4 times per week** \| **Most days** \| **Daily** \| \| **In the past week, how often have you…** \| \| \| \| \| \| \| Encouraged your child to participate in physical activity or play **outdoors** \|  \|  \|  \|  \|  \| \| Engaged in physical activity or play **outdoors** with your child \|  \|  \|  \|  \|  \| \| Provided transportation to a place where your child can be active **outdoors** \|  \|  \|  \|  \|  \| \| Watched your child participate in physical activity or play **outdoors** \|  \|  \|  \|  \|  \| \| Told your child being active **outdoors** was good for their health \|  \|  \|  \|  \|  \| | | | |
|  | The following questions ask you to rate how you feel about supporting your child’s active play and physical activity **outdoors**. | | | |
| **60.** | For me, regularly supporting my child’s active **outdoor** play over the next week would be:   \|  \| **1 (extremely disagree)** \| **2** \| **3** \| **4 (neutral)** \| **5** \| **6** \| **7 (extremely agree)** \| \| --- \| --- \| --- \| --- \| --- \| --- \| --- \| --- \| \| Enjoyable \|  \|  \|  \|  \|  \|  \|  \| \| Wise \|  \|  \|  \|  \|  \|  \|  \| \| Exciting \|  \|  \|  \|  \|  \|  \|  \| \| Beneficial \|  \|  \|  \|  \|  \|  \|  \| \| Useful \|  \|  \|  \|  \|  \|  \|  \| \| Pleasant \|  \|  \|  \|  \|  \|  \|  \| | | | |
