# Supplementary material for: Effect of an Outdoor-Focused Licensed Child Care Program on Child, Caregiver, and Educator Outcomes, Inclusion, and Accessibility: Protocol for the Sending Preschoolers Outside (SPROUT) Prospective Cohort Study
Source: JMIR Res Protoc. 2026 Jul 21;15:e89405. doi: 10.2196/89405 (PMC13387637; doi:10.2196/89405)
Supplement: Multimedia Appendix 2 [file resprot-v15-e89405-s002.docx]

**Appendix B: Educator Questionnaire**

| **Part 1: About You** | |
| --- | --- |
| **1.** | **How old are you (in years)?** |
|  | _______ years |
| **2.** | **What was your sex at birth?** |
|  | - Female - Male - Intersex - Unknown - Prefer not to answer |
| **3.** | **What is your current gender identity?**   - Female - Male - Another Gender   What gender do you identify as? ___________________   - Unknown - Not applicable - Prefer not to answer |
| **4.** | **In our society, people are often described by their race or racial background. These are not based in science, but our race may influence the way we are treated by individuals and institutions, and this may affect our health. Which category(ies) best describes you? Check all that apply:** |
|  | - Black (e.g., African, African Canadian, Afro-Caribbean descent) - East Asian (e.g., Chinese, Japanese, Korean, Taiwanese descent) - Indigenous (e.g., First Nations, Inuk/Inuit, Métis descent) - Latin American (e.g., Hispanic or Latin American descent) - Middle Eastern (e.g., Arab, Persian, West Asian [e.g., Afghan, Egyptian, Iranian, Kurdish, Lebanese, Turkish] descent) - South Asian (e.g., Bangladeshi, Indian, Indo-Caribbean, Pakistani, Sri Lankan descent) - Southeast Asian (e.g., Cambodian, Filipino, Indonesian, Thai, Vietnamese descent) - White (e.g., European descent) - Do not know - Prefer not to answer - Another race category (Optional - please specify:) _______________________________ |
| **5.** | **How many years have you been a practicing RECE?** |
|  | _______ years |
| **6.** | **Have you ever worked with children with disabilities (e.g., physical disabilities, developmental delay, cognitive disabilities, sensory impairments)?** |
|  | - Yes - How many years’ experience do you have working with children with disabilities? _______   No |
| **7a.** | **Have you ever worked at a nature-based childcare centre?** |
|  | - Yes - How many years’ experience do you have working in a nature-based childcare centre? _______ - No |
| **7b.** | **If yes to Question 7a: Do you have experience working with children with disabilities (e.g., physical disabilities, developmental delay, cognitive disabilities, sensory impairments) in a a nature-based childcare centre, or more generally in an outdoor setting?** |
|  | - Yes - How many years’ experience do you have working with children with disabilities in a nature-based childcare centre or outdoor setting? _______ - No |
| **9.** | **What is the highest level of education you have completed?** |
|  | - Secondary or high school - Vocational/College education - Tertiary/University education - Prefer not to answer |
|  |  |
| **Part 2: Job Satisfaction, Health and Wellbeing** | |
| **10.** | **Regarding your work in general. How pleased are you with …**   \|  \| Very unsatisfied \| Unsatisfied \| Neither/nor \| Satisfied \| Very satisfied \| \| --- \| --- \| --- \| --- \| --- \| --- \| \| Your work prospects? \|  \|  \|  \|  \|  \| \| The physical working conditions? \|  \|  \|  \|  \|  \| \| The way your abilities are used? \|  \|  \|  \|  \|  \| \| Your job as a whole, everything taken into consideration? \|  \|  \|  \|  \|  \| \| Your salary? \|  \|  \|  \|  \|  \| |
|  | **The following questions are about your own health and well-being. Please do not try to distinguish between symptoms that are caused by work and symptoms that are due to other causes. The task is to describe how you are in general.**  **The questions are about your health and well-being during the last four weeks:** |
| **11.** | **In general, would you say your health is:**   - Excellent - Very good - Good - Fair - Poor |
|  | **These questions are about how you have been during the last 4 weeks.**   \|  \| Not at all \| A small part of the time \| Part of the time \| A large part of the time \| All the time \| \| --- \| --- \| --- \| --- \| --- \| --- \| \| **How often have you…** \| \| \| \| \| \| \| Slept badly and restlessly? \|  \|  \|  \|  \|  \| \| Found it hard to go to sleep? \|  \|  \|  \|  \|  \| \| Woken up too early and not been able to get back to sleep? \|  \|  \|  \|  \|  \| \| Woken up several times and found it difficult to get back to sleep? \|  \|  \|  \|  \|  \| |
| **12.** | **These questions are about how you have been during the last 4 weeks.**   \|  \| Not at all \| A small part of the time \| Part of the time \| A large part of the time \| All the time \| \| --- \| --- \| --- \| --- \| --- \| --- \| \| **How often have you…** \| \| \| \| \| \| \| Felt worn out? \|  \|  \|  \|  \|  \| \| Been physically exhausted? \|  \|  \|  \|  \|  \| \| Been emotionally exhausted? \|  \|  \|  \|  \|  \| \| Felt tired? \|  \|  \|  \|  \|  \| |
| **13.** | **These questions are about how you have been during the last 4 weeks**.   \|  \| Not at all \| A small part of the time \| Part of the time \| A large part of the time \| All the time \| \| --- \| --- \| --- \| --- \| --- \| --- \| \| **How often have you…** \| \| \| \| \| \| \| Had problems relaxing? \|  \|  \|  \|  \|  \| \| Been irritable? \|  \|  \|  \|  \|  \| \| Been tense? \|  \|  \|  \|  \|  \| |
| **14.** | **These questions are about how you have been during the last 4 weeks.**   \|  \| Not at all \| A small part of the time \| Part of the time \| A large part of the time \| All the time \| \| --- \| --- \| --- \| --- \| --- \| --- \| \| **How often have you…** \| \| \| \| \| \| \| Had a stomach-ache? \|  \|  \|  \|  \|  \| \| Had a headache? \|  \|  \|  \|  \|  \| \| Had palpitations? \|  \|  \|  \|  \|  \| \| Had tension in various muscles? \|  \|  \|  \|  \|  \| |
| **15.** | **These questions are about how you have been during the last 4 weeks.**   \|  \| Not at all \| A small part of the time \| Part of the time \| A large part of the time \| All the time \| \| --- \| --- \| --- \| --- \| --- \| --- \| \| **How often have you…** \| \| \| \| \| \| \| Had problems concentrating? \|  \|  \|  \|  \|  \| \| Found it difficult to think clearly? \|  \|  \|  \|  \|  \| \| Had difficulty in making decisions? \|  \|  \|  \|  \|  \| \| Had difficulty remembering? \|  \|  \|  \|  \|  \| |
| **16.** | **These questions are about how you have been during the last 4 weeks.**   \|  \| Not at all \| A small part of the time \| Part of the time \| A large part of the time \| All the time \| \| --- \| --- \| --- \| --- \| --- \| --- \| \| **How often have you…** \| \| \| \| \| \| \| Felt sad? \|  \|  \|  \|  \|  \| \| Lacked self-confidence? \|  \|  \|  \|  \|  \| \| A bad conscience or felt guilty? \|  \|  \|  \|  \|  \| \| Lacked interest in everyday things? \|  \|  \|  \|  \|  \| |
| **17.** | **How well do these descriptions fit you as a person?**   \|  \| Does not fit \| Fits a little \| Fits quite well \| Fits perfectly \| \| --- \| --- \| --- \| --- \| --- \| \| I am always able to solve difficult problems, if I try hard enough. \|  \|  \|  \|  \| \| If people work against me, I find a way of achieving what I want \|  \|  \|  \|  \| \| It is easy for me to stick to my plans and reach my objectives \|  \|  \|  \|  \| \| I feel confident that I can handle unexpected events. \|  \|  \|  \|  \| \| When I have a problem, I can usually find several ways of solving it \|  \|  \|  \|  \| \| Regardless of what happens, I usually manage. \|  \|  \|  \|  \| |

**18.**

The following questions are about your confidence to perform various activities during the childcare day in general, **among children without a disability or complex needs** (e.g., developmental disability, low vision, mobility impairments).

Please indicate on a scale of 0 (not confident at all) to 10 (completely confident), **how confident you are in your general ability to perform the following** during your childcare day:

| **Promoting Physical Activity** | | | | | | | | | | | |
| --- | --- | --- | --- | --- | --- | --- | --- | --- | --- | --- | --- |
|  | **0** | **1** | **2** | **3** | **4** | **5** | **6** | **7** | **8** | **9** | **10** |
|  | Not confident at all |  |  |  |  | Moderately confident |  |  |  |  | Completely confident |
| Program opportunities for at least 120 min/day of physical activity at any intensity |  |  |  |  |  |  |  |  |  |  |  |
| Facilitate higher intensity physical activity (i.e., activities that induce sweating and heavy breathing) for children in my care everyday |  |  |  |  |  |  |  |  |  |  |  |
| Lead activities that promote children’s development of physical literacy (e.g., including running, jumping, balancing, throwing, catching) |  |  |  |  |  |  |  |  |  |  |  |
| Adapt physical activities for different developmental abilities |  |  |  |  |  |  |  |  |  |  |  |
| Support children’s motivation to move through verbal encouragement |  |  |  |  |  |  |  |  |  |  |  |
| Incorporate movement into your curriculum (e.g., literacy and numeracy) |  |  |  |  |  |  |  |  |  |  |  |
| Lead structured (i.e., educator-facilitated) physical activity each day |  |  |  |  |  |  |  |  |  |  |  |
| Teach children about the health benefits of physical activity |  |  |  |  |  |  |  |  |  |  |  |
| Communicate about children’s physical activity and physical literacy with families |  |  |  |  |  |  |  |  |  |  |  |
| Develop organizational policies for physical activity |  |  |  |  |  |  |  |  |  |  |  |
| Serve as a positive role model for children’s physical activity by participating in movement-based activities |  |  |  |  |  |  |  |  |  |  |  |
| Lead structured physical activities in a small/limited space |  |  |  |  |  |  |  |  |  |  |  |
| Encourage physical activity even when my colleagues/superiors do not value it |  |  |  |  |  |  |  |  |  |  |  |
| Program opportunities for physical activity even when there are time constraints |  |  |  |  |  |  |  |  |  |  |  |

| **Promoting Active Play** | | | | | | | | | | | |
| --- | --- | --- | --- | --- | --- | --- | --- | --- | --- | --- | --- |
|  | **0** | **1** | **2** | **3** | **4** | **5** | **6** | **7** | **8** | **9** | **10** |
|  | Not confident at all |  |  |  |  | Moderately confident |  |  |  |  | Completely confident |
| Facilitate opportunities for unstructured (i.e., child-directed) active play each day |  |  |  |  |  |  |  |  |  |  |  |
| Create an environment that supports children’s active play |  |  |  |  |  |  |  |  |  |  |  |
| Engage children in age-appropriate risky play (i.e., adventurous play that tests children’s limits but involves a chance of minor injury; e.g., play at heights, high speeds) |  |  |  |  |  |  |  |  |  |  |  |
| Provide children with multiple outdoor play opportunities everyday |  |  |  |  |  |  |  |  |  |  |  |
| Provide outdoor playtime even when the weather conditions are not favourable, but not extreme |  |  |  |  |  |  |  |  |  |  |  |
| Facilitate children’s active play indoors when outdoor play is not an option or extreme weather prevents outdoor play |  |  |  |  |  |  |  |  |  |  |  |
| Facilitate opportunities for age-appropriate risky play even when met with resistance from parents/ other educators |  |  |  |  |  |  |  |  |  |  |  |
| Facilitate outdoor active play opportunities even when I am tired |  |  |  |  |  |  |  |  |  |  |  |
| Facilitate outdoor active play opportunities even when transitions are difficult |  |  |  |  |  |  |  |  |  |  |  |
| Facilitate outdoor active play opportunities even when it takes additional time to get children dressed |  |  |  |  |  |  |  |  |  |  |  |

| **Reducing Sedentary Time** | | | | | | | | | | | |
| --- | --- | --- | --- | --- | --- | --- | --- | --- | --- | --- | --- |
|  | **0** | **1** | **2** | **3** | **4** | **5** | **6** | **7** | **8** | **9** | **10** |
|  | Not confident at all |  |  |  |  | Moderately confident |  |  |  |  | Completely confident |
| Develop organizational policies for screen time |  |  |  |  |  |  |  |  |  |  |  |
| Serve as a positive role model for children’s sedentary behaviours by limiting your own sitting during care hours |  |  |  |  |  |  |  |  |  |  |  |
| Serve as a positive role model for children’s screen behaviours by limiting your own screen use during care hours |  |  |  |  |  |  |  |  |  |  |  |
| Minimize long periods (>60 minutes) of sitting time among the children in my care |  |  |  |  |  |  |  |  |  |  |  |
| Incorporate physical activity opportunities (e.g., active breaks and transitions) to minimize prolonged sitting time |  |  |  |  |  |  |  |  |  |  |  |
| Avoid children’s use of screen-based technology during childcare hours |  |  |  |  |  |  |  |  |  |  |  |
| Minimize children’s sedentary behaviour when they are tired |  |  |  |  |  |  |  |  |  |  |  |

**19.**

The following questions are about your confidence to perform various activities during the childcare day **specifically among children with a disability and/or complex needs (e.g., developmental disability, low vision, mobility impairments).**

Please indicate on a scale of 0 (not confident at all) to 10 (completely confident), **how confident you are in your ability to perform the following** during your childcare day among children with disabilities and/or complex needs **(e.g., developmental disability, low vision, mobility impairments)**:

| **Promoting Physical Activity** | | | | | | | | | | | |
| --- | --- | --- | --- | --- | --- | --- | --- | --- | --- | --- | --- |
|  | **0** | **1** | **2** | **3** | **4** | **5** | **6** | **7** | **8** | **9** | **10** |
|  | Not confident at all |  |  |  |  | Moderately confident |  |  |  |  | Completely confident |
| Program opportunities for at least 120 min/day of physical activity at any intensity |  |  |  |  |  |  |  |  |  |  |  |
| Facilitate higher intensity physical activity (i.e., activities that induce sweating and heavy breathing) for children in my care everyday |  |  |  |  |  |  |  |  |  |  |  |
| Lead activities that promote children’s development of physical literacy (e.g., including running, jumping, balancing, throwing, catching) |  |  |  |  |  |  |  |  |  |  |  |
| Adapt physical activities for different developmental abilities |  |  |  |  |  |  |  |  |  |  |  |
| Support children’s motivation to move through verbal encouragement |  |  |  |  |  |  |  |  |  |  |  |
| Incorporate movement into your curriculum (e.g., literacy and numeracy) |  |  |  |  |  |  |  |  |  |  |  |
| Lead structured (i.e., educator-facilitated) physical activity each day |  |  |  |  |  |  |  |  |  |  |  |
| Teach children about the health benefits of physical activity |  |  |  |  |  |  |  |  |  |  |  |
| Communicate about children’s physical activity and physical literacy with families |  |  |  |  |  |  |  |  |  |  |  |
| Develop organizational policies for physical activity |  |  |  |  |  |  |  |  |  |  |  |
| Serve as a positive role model for children’s physical activity by participating in movement-based activities |  |  |  |  |  |  |  |  |  |  |  |
| Lead structured physical activities in a small/limited space |  |  |  |  |  |  |  |  |  |  |  |
| Encourage physical activity even when my colleagues/superiors do not value it |  |  |  |  |  |  |  |  |  |  |  |
| Program opportunities for physical activity even when there are time constraints |  |  |  |  |  |  |  |  |  |  |  |

| **Promoting Active Play** | | | | | | | | | | | |
| --- | --- | --- | --- | --- | --- | --- | --- | --- | --- | --- | --- |
|  | **0** | **1** | **2** | **3** | **4** | **5** | **6** | **7** | **8** | **9** | **10** |
|  | Not confident at all |  |  |  |  | Moderately confident |  |  |  |  | Completely confident |
| Facilitate opportunities for unstructured (i.e., child-directed) active play each day |  |  |  |  |  |  |  |  |  |  |  |
| Create an environment that supports children’s active play |  |  |  |  |  |  |  |  |  |  |  |
| Engage children in age-appropriate risky play (i.e., adventurous play that tests children’s limits but involves a chance of minor injury; e.g., play at heights, high speeds) |  |  |  |  |  |  |  |  |  |  |  |
| Provide children with multiple outdoor play opportunities everyday |  |  |  |  |  |  |  |  |  |  |  |
| Provide outdoor playtime even when the weather conditions are not favourable, but not extreme |  |  |  |  |  |  |  |  |  |  |  |
| Facilitate children’s active play indoors when outdoor play is not an option or extreme weather prevents outdoor play |  |  |  |  |  |  |  |  |  |  |  |
| Facilitate opportunities for age-appropriate risky play even when met with resistance from parents/ other educators |  |  |  |  |  |  |  |  |  |  |  |
| Facilitate outdoor active play opportunities even when I am tired |  |  |  |  |  |  |  |  |  |  |  |
| Facilitate outdoor active play opportunities even when transitions are difficult |  |  |  |  |  |  |  |  |  |  |  |
| Facilitate outdoor active play opportunities even when it takes additional time to get children dressed |  |  |  |  |  |  |  |  |  |  |  |

| **Reducing Sedentary Time** | | | | | | | | | | | |
| --- | --- | --- | --- | --- | --- | --- | --- | --- | --- | --- | --- |
|  | **0** | **1** | **2** | **3** | **4** | **5** | **6** | **7** | **8** | **9** | **10** |
|  | Not confident at all |  |  |  |  | Moderately confident |  |  |  |  | Completely confident |
| Develop organizational policies for screen time |  |  |  |  |  |  |  |  |  |  |  |
| Serve as a positive role model for children’s sedentary behaviours by limiting your own sitting during care hours |  |  |  |  |  |  |  |  |  |  |  |
| Serve as a positive role model for children’s screen behaviours by limiting your own screen use during care hours |  |  |  |  |  |  |  |  |  |  |  |
| Minimize long periods (>60 minutes) of sitting time among the children in my care |  |  |  |  |  |  |  |  |  |  |  |
| Incorporate physical activity opportunities (e.g., active breaks and transitions) to minimize prolonged sitting time |  |  |  |  |  |  |  |  |  |  |  |
| Avoid children’s use of screen-based technology during childcare hours |  |  |  |  |  |  |  |  |  |  |  |
| Minimize children’s sedentary behaviour when they are tired |  |  |  |  |  |  |  |  |  |  |  |

**19.**

The following questions ask you to rate **how you feel** about supporting children’s active play and physical activity **outdoors** at your childcare centre.

For me, regularly supporting active **outdoor** play for children **without** a disability or complex needs at my childcare centre over the next week would be:

|  | **1 (extremely disagree)** | **2** | **3** | **4 (neutral)** | **5** | **6** | **7 (extremely agree)** |
| --- | --- | --- | --- | --- | --- | --- | --- |
| Enjoyable |  |  |  |  |  |  |  |
| Wise |  |  |  |  |  |  |  |
| Exciting |  |  |  |  |  |  |  |
| Beneficial |  |  |  |  |  |  |  |
| Useful |  |  |  |  |  |  |  |
| Pleasant |  |  |  |  |  |  |  |

**20.**

For me, regularly supporting children’s active **outdoor** play at my childcare centre for children **with a disability or complex needs** over the next week would be:

|  | **1 (extremely disagree)** | **2** | **3** | **4 (neutral)** | **5** | **6** | **7 (extremely agree)** |
| --- | --- | --- | --- | --- | --- | --- | --- |
| Enjoyable |  |  |  |  |  |  |  |
| Wise |  |  |  |  |  |  |  |
| Exciting |  |  |  |  |  |  |  |
| Beneficial |  |  |  |  |  |  |  |
| Useful |  |  |  |  |  |  |  |
| Pleasant |  |  |  |  |  |  |  |

21. Reflecting on your practice, what supports/resources have been key to you gaining confidence, competence, and/or comfort leading outdoor play for children of all abilities in the early years?
