# Supplementary material for: Effect of an Outdoor-Focused Licensed Child Care Program on Child, Caregiver, and Educator Outcomes, Inclusion, and Accessibility: Protocol for the Sending Preschoolers Outside (SPROUT) Prospective Cohort Study
Source: JMIR Res Protoc. 2026 Jul 21;15:e89405. doi: 10.2196/89405 (PMC13387637; doi:10.2196/89405)
Supplement: Multimedia Appendix 3 [file resprot-v15-e89405-s003.docx]

**Appendix C: Injury Statistics Questionnaire for Childcare Centres**

1. **Number of children enrolled in your program(s)?**

**_______**

1. **Age of the children in your program(s) (select all that apply)**
   - 0-2
   - 2-3
   - 3-4
   - 5+
2. **On an average program day, how long is (are) the program(s) you lead (in hours)?**

**_______**

1. **On an average program day with normal weather conditions, how many hours do educators outdoors with the children in your program(s)?**

**_______**

1. **On an average program day with unfavorable weather conditions (i.e., rain, snow, cold...), how many hours do educators spend outdoors with the children in your program(s), if any?**

**_______**

1. **When taking the children outside, where do educators usually go?** (select all that apply)
   - Playground
   - Public Park
   - Forest
   - Farm
   - Field
   - Urban wooded areas
   - Childcare playground as part of your centre
   - Public school playground
2. **Are there any additional details you would like to share with us?**
3. **In the last year what was the number of low intensity injuries at your centre overall?**
   - - Low intensity injuries require minor first aid attention (e.g., Minor Wounds, Minor Cuts, Scrapes, Nosebleeds, etc.)
4. **If available, what activities were the children involved in during these injuries?** (e.g., climbing, running, playing with an object; please indicate activity and number of associated injuries, if possible)
5. **If available, what surfaces were the children on during these injuries?** (e.g., pavement, sand; please indicate surface and number of associated injuries, if possible)
6. **How many of those injuries happened outdoors?**
7. **How many of those injured were boys?**
8. **In the last year what was the number of medium intensity injuries at your centre overall?**
   - - Medium intensity injuries require medical attention (e.g., larger cuts requiring stitches, small fractures or sprains, temporary loss of consciousness, etc.)
9. **If available, what activities were the children involved in during these injuries?** (e.g., climbing, running, playing with an object; please indicate activity and number of associated injuries, if possible)
10. **If available, what surfaces were the children on during these injuries?** (e.g., pavement, sand; please indicate surface and number of associated injuries, if possible)
11. **How many of those injuries happened outdoors?**
12. **How many of those injured were boys?**
13. **In the last year what was the number of high intensity injuries at your centre overall?**
    - - High intensity injuries require immediate and serious medical attention (e.g., broken bones, serious head injuries, internal bleeding, or other lethal and near-lethal experiences)
14. **If available, what activities were the children involved in during these injuries?** (e.g., climbing, running, playing with an object; please indicate activity and number of associated injuries, if possible)
15. **If available, what surfaces were the children on during these injuries?** (e.g., pavement, sand; please indicate surface and number of associated injuries, if possible)
16. **How many of those injuries happened outdoors?**
17. **How many of those injured were boys?**
18. **Are there any additional details you would like to share with us?**
