# Supplementary material for: Effect of an Outdoor-Focused Licensed Child Care Program on Child, Caregiver, and Educator Outcomes, Inclusion, and Accessibility: Protocol for the Sending Preschoolers Outside (SPROUT) Prospective Cohort Study
Source: JMIR Res Protoc. 2026 Jul 21;15:e89405. doi: 10.2196/89405 (PMC13387637; doi:10.2196/89405)
Supplement: Multimedia Appendix 4 [file resprot-v15-e89405-s004.docx]

| **SPROUT-able Involvement Matrix** | | | **Role in Project** | | | |
| --- | --- | --- | --- | --- | --- | --- |
|  |  |  | **Co-Thinker**  Is asked to give opinion  (How do you think this sounds?) | **Advisor**  Gives advice  (How would you approach this?) | **Partner**  Works as a partner  (Can you work with me on this?) | **Decision Maker**  Takes initiative and drives decision making  (Can you complete this?) |
| **Stage of Project Research** | Preparation | **Developing research questions (RQ)** | Provides feedback on the developed RQ | Provides advice on how to develop a RQ with options or examples | Develops the RQ alongside a researcher | Develops the RQ independently* and shares back with the team for feedback and further development |
|  |  | **Developing interview guides for focus groups** | Reviewing and *adding comments* to the interview guides | Reviewing and *editing* the interview guides | Developing a portion of the interview guide* | Creating the interview guide* and sharing back with the team for feedback |
|  |  | **Developing data collection protocol** | Giving opinions to the data collection procedures (e.g., Do you think these procedures are feasible? Do you think we are missing anything in our protocol? Are the protocols accessible and inclusive?) | Providing ideas for the protocol (i.e., what are some things we should include in our n protocol? What should we me assessing/observing? How should we assess/observe this? How do we ensure inclusive participation in the research study?) | Working closely with a team member on developing the protocol | Co-developing the protocol* - may develop parts of the protocol independently |
|  |  | **Recruitment** | Giving opinions to the recruitment strategy (e.g., do you think we are effectively reaching our population of interest? Are we missing groups of individuals with our recruitment strategy? Are our recruitment strategies accessible and inclusive?) | Providing ideas for how to recruit participants and engage participants meaningfully in the research project (e.g., single meeting) | Helping to develop a general recruitment strategy with members of the team (e.g., multiple meetings) | Developing a recruitment strategy for participants within your networks independently or alongside a team member |
|  | Execution | **Interviews and Focus Groups** | Give opinion on the structure of the focus groups and how the focus groups can be more accessible and inclusive | Provide advice on the structure and setup of the focus groups and tangible strategies for implementing the focus group (e.g., location, number of participants) | Attend the focus group, taking notes during the focus group, and provide support to the lead interviewer | Conduct portions of the focus group alongside a team member* |
|  |  | **Data Collection** | Meet with data collection staff for updates and provide opinion on data collection progress (e.g., if data collectors are facing difficulty in an aspect of data collection, providing opinion for how to make improvements) | Meet with data collection staff for updates and help determine strategies for addressing challenges with data collection (this could involve shadowing study appointments if it is of interest) | Attending data collection sessions to observe and support the data collector | Attend data collection sessions to help conduct data collection* |
|  |  | **Analyzing Data** | Reviewing findings and providing opinion | Attend data analysis meetings to provide ideas and suggestions for data analysis | Assist with interpretation of the findings, may participate in data analysis as a mentee* | Complete a portion of data analysis and lead sections of the data interpretation* |
|  | Disseminate and Report | **Manuscript Writing**  **(May include plain language abstract writing)** | Reviewing and *adding comments* to the manuscript | Reviewing and *editing* the manuscript | Writing a lay summary of the manuscript *  i.e., plain language abstract | Write a portion of the manuscript* to share back with the team. |
|  |  | **Presenting Findings (e.g., conferences, community meetings)** | Reviewing and *adding comments* to presentation material (e.g., slides, presentation notes) | Reviewing and *editing* the presentation material | Developing parts of the presentation material, may also have opportunity to attend events* | Presenting and/or co-presenting at events* |
|  |  | **Knowledge Products** | Reviewing and *adding comments* to knowledge products | Provide ideas for knowledge products, identify gaps for new resources, dissemination of knowledge products | Developing knowledge products with a team member, supporting the development of a knowledge mobilization plan* | Creating knowledge products and mobilization plan independently or leading a team* |
| *training and/or mentorship will be provided when needed | | | | | | |
